# A Putative Type II Secretion System Is Involved in Cellulose Utilization in *Cytophaga hutchisonii*

**DOI:** 10.3389/fmicb.2017.01482

**Published:** 2017-08-09

**Authors:** Xia Wang, Qingqing Han, Guanjun Chen, Weixin Zhang, Weifeng Liu

**Affiliations:** State Key Laboratory of Microbial Technology, School of Life Science, Shandong University Jinan, China

**Keywords:** *Cytophaga hutchisonii*, cellulose degradation, cellulose adhesion, protein secretion, T2SS

## Abstract

*Cytophaga hutchinsonii* is a gliding cellulolytic bacterium that degrades cellulose in a substrate contact-dependent manner. Specific proteins are speculated to be translocated to its extracellular milieu or outer membrane surface to participate in adhesion to cellulose and further digestion. In this study, we show that three orthologous genes encoding the major components (T2S-D, -F, and -G) of type II secretion system (T2SS) are involved in cellulose degradation but not in cell motility. The individual disruption of the three *t2s* genes results in a significantly retarded growth on cellobiose, regenerated amorphous cellulose, and Avicel cellulose. Enzymatic analyses demonstrate that, whereas the endoglucanase activity of the *t2s* mutant cells is increased, the β-glucosidase activity is remarkably reduced compared to that of WT cells. Further analyses reveal that the *t2s* mutant cells not only exhibit a different profile of cellulose-bound outer membrane proteins from that of wild-type cells, but also display a significant decrease in their capability to adhere to cellulose. These results indicate that a functional link exits between the putative T2SS and cellulose utilization in *C. hutchinsonii*, and thus provide a conceptual framework to understand the unique strategy deployed by *C. hutchinsonii* to assimilate cellulose.

## Introduction

*Cytophaga hutchinsonii* is a common cellulolytic soil bacterium belonging to the phylum *Bacteroidetes* (Stanier, 1942; Reichenbach, 2006; Xie et al., 2007). *C. hutchinsonii* cells are capable of actively digesting crystalline cellulose in a substrate contact-dependent manner (Stanier, 1942; Larkin, 1989). Unlike most other well-studied cellulolytic microorganisms which apply either the extracellular free cellulase system or the cell surface-anchored multiprotein cellulosome to achieve the efficient degradation of cellulose (Lynd et al., 2002), *C. hutchinsonii* has been postulated to use a third but poorly understood strategy to digest cellulose (Wilson, 2008). Analysis of *C. hutchinsonii* genomic sequences reveals that no obvious homologs of cellobiohydrolases are present and all the annotated endoglucanases lack recognizable cellulose binding modules (CBMs; Xie et al., 2007). Although, several processive endoglucanases that may act as functional equivalents of exocellulases have been described (Ji et al., 2012; Zhu et al., 2013; Zhang et al., 2014), it has been recently found that only two periplasmic non-processive endoglucanases are critical for *C. hutchinsonii* growth on cellulose (Zhu et al., 2016). Additionally, since no genes encoding proteins with type A CBM that mediate binding to crystalline cellulose are present in *C. hutchinsonii* genome, adhesion of *C. hutchinsonii* to cellulose also appears to involve a novel set of cellulose-binding proteins (CBPs) located on the outer membrane (Gong and Forsberg, 1989; Jun et al., 2007).

Like many members in the phylum *Bacteroidetes*, another distinct feature of *C. hutchinsonii* is its rapid gliding motility over surfaces which has been speculated to facilitate cellulose assimilation by allowing cells to access regions of cellulose more amenable to digestion (Stanier, 1942). *Bacteroidetes* gliding motility has been extensively studied in the distantly related *Flavobacterium johnsoniae* where 19 Gld and Spr proteins involved in motility have been identified (Braun et al., 2005; McBride et al., 2009; Nakane et al., 2013; Shrivastava et al., 2013). Moreover, some Gld and Spr proteins have even been considered to be components of the recently established type IX secretion system (T9SS), previously referred to as the Por secretion system (McBride and Zhu, 2013; Shrivastava et al., 2013). T9SS genes appear to be present in many members of the phylum *Bacteroidetes* including *C. hutchinsonii*, which possesses genes predicted to encode orthologs of each of the T9SS proteins and presumably uses the system for protein secretion (Xie et al., 2007; McBride and Zhu, 2013). The functional involvement of T9SS in *C. hutchinsonii* cellulose utilization has been demonstrated by the observation that the individual absence of two T9SS components, SprP and PorU, results in defects in gliding and cellulose degradation (Wang et al., 2014; Zhu and McBride, 2014).

Besides T9SS, prokaryotes have evolved many ways of transporting protein cargo between locations (Green and Mecsas, 2016). One important system used by many Gram-negative bacteria to translocate proteins from the periplasm to the extracellular milieu or outer membrane surface is the type II secretion system (T2SS) (Korotkov et al., 2012; Rondelet and Condemine, 2013; Cianciotto and White, 2017). The T2SS is a sophisticated multiprotein machinery proposed to span both the inner and outer membranes which can be further subdivided into four subassemblies including the pseudopilus, the outer-membrane complex, the inner-membrane platform, and the secretion ATPase (Sandkvist, 2001; Korotkov et al., 2012), for which the 12–15 component proteins are generally encoded in a single operon. While the terminology GSP (general secretory pathway) has been sometimes used to referred to the different components of the T2S apparatus, its use needs to be refrained and in any case restricted solely to the description of translocation through the cytoplasmic membrane via the Sec apparatus and not to the components of the T2SS *per se* (Desvaux et al., 2004). It has also become apparent that the T2SS and the type 4 pilus system (T4PS) are evolutionarily related and share several structural and functional features (Peabody et al., 2003; Korotkov et al., 2012; Nivaskumar and Francetic, 2014). Although, genomic analysis revealed no homologous genes encoding the typical type 4 pilins, four genes encoding putative T2S-D (also called PulD or XcpQ in *Klebsiella oxytoca* and *Pseudomonas aeruginosa* respectively, and formerly GspD), T2S-E (also called PulE or XcpR, and formerly GspE), T2S-F (also called PulF or XcpS, and formerly GspS), and T2S-G (also called PulG or XcpT, and formerly GspG) of T2SS have been identified in *C. hutchinsonii* (Peabody et al., 2003; Cianciotto, 2005; McBride and Zhu, 2013; Cianciotto and White, 2017). Here we constructed chromosomal insertions or deletions for three of these *t2s* genes. These *t2s* mutants were found to be deficient in cellulose degradation but not in gliding motility, suggesting a link between a putative T2SS and cellulose utilization in *C. hutchinsonii*.

## Materials and methods

### Bacterial strains, plasmids, and culture conditions

Strains and plasmids used in this study were listed in Table 1. *C. hutchinsonii* ATCC 33406 was used as the wild-type strain (WT) throughout this work. WT and mutant strains were maintained at 28°C in liquid or on solid PY10 medium containing 1.0% peptone, 0.05% yeast extract at pH 7.3, supplemented with different carbon sources as indicated. *Escherichia coli* was routinely cultured in Luria-Bertani medium supplemented with the following antibiotics when necessary: ampicillin, 100 μg/ml; kanamycin, 40 μg/ml; erythromycin, 60 μg/ml, and chloramphenicol, 15 μg/ml.

**Table 1:** Strains and plasmids used in this study.

**Strains and plasmids**	**Description**	**References or sources**
**STRAINS**		
*C. hutchinsonii*	wild type	ATCC
ATCC 33406		
Δ3195	Targeted insertion in *chu_3195*; *Em^*r*^*	This study
Δ3196	Targeted insertion in *chu_3196*; *Em^*r*^*	This study
Δ3198	Targeted deletion of *chu_3198*; *Em^*r*^*	This study
Δ3199	Targeted insertion in *chu_3199*; *Em^*r*^*	This study
Δ1253	Targeted insertion in *chu_1253*; *Em^*r*^*	This study
COM3195	Complemented strain with plasmid pCH3195; *Em^*r*^, Cm^*r*^*	This study
COM3199	Complemented strain with plasmid pCH3199; *Em^*r*^, Cm^*r*^*	This study
*E. coli* DH5α	F−φ80*lacZ*ΔM15 Δ*(lacZYA-argF)* U169 *recA1 endA1 hsdR17(rk^−^, mk^+^) phoA supE44 λ− thi-1 gyrA96 relA1*	Laboratory stock
**PLASMIDS^a^**		
pLYL03	ColE1; *Bacteroides*–*Flavobacterium* suicide vector; *Ap^*r*^* (*Em^*r*^*)	Li et al., 1995
pYT313	*sacB*-containing suicide vector; *Ap^*r*^ (Em^*r*^)*	
pLYIN3195	pLYL03 carrying an 1.0-kbp internal fragment of *chu_1719*; *Ap^*r*^* (*Em^*r*^*)	This study
pYT3198	PYT313 carrying two 2.0-kbp fragments upstream and downstream of *chu_3198*; *Ap^*r*^* (*Em^*r*^*)	This study
pLYIN3199	pLYL03 carrying an 748-bp internal fragment of *chu_3196*; *Ap^*r*^* (*Em^*r*^*)	This study
pLYIN1253	pLYL03 carrying an 825-bp internal fragment of *chu_1253*; *Ap^*r*^* (*Em^*r*^*)	This study
pCH03C	pLYL03oriC containing *cat* resistant gene; *Ap^*r*^* (*Em^*r*^, Cm^*r*^*)	Zhou et al., 2016
pCH3195	pCH03C containing an expression cassette of *chu_3195* under control of the *chu_1284* promoter; *Ap^*r*^* (*Em^*r*^, Cm^*r*^*)	This study
pCH3198	pCH03C containing an expression cassette of *chu_3198* under control of the *PompA* promoter; *Ap^*r*^* (*Em^*r*^, Cm^*r*^*)	This study
pCH3199	pCH03C containing an expression cassette of *chu_3199* under control of the *chu_1284* promoter; *Ap^*r*^* (*Em^*r*^, Cm^*r*^*)	This study

a *Antibiotic resistance phenotypes: ampicillin (Ap^r^), chloramphenicol (Cm^r^), erythromycin (Em^r^), kanamycin (Km^r^). Unless indicated otherwise, the antibiotic resistance phenotypes are those expressed in E. coli. The antibiotic resistance phenotypes in parentheses are expressed in C. hutchinsonii*.

### Plasmid constructions

An 1,000-bp fragment within the CHU_3195 coding frame was amplified from *C. hutchinsonii* chromosomal DNA with primers 3195inF and 3195inR, and then ligated into the plasmid pLYL03 digested by *Bam*HI and *Sal*I to generate the pLYIN3195 plasmid. Similarly, an 800-bp fragment within the CHU_3199 coding frame was amplified from *C. hutchinsonii* chromosomal DNA with primers 3199inF and 3199inR, and inserted into the *Bam*HI/*Sal*I sites within the pLYL03 plasmid to obtain the pLYIN3199 plasmid. To construct the pLYIN1253 plasmid, an 825-bp fragment within CHU_1253 coding frame was also amplified from *C. hutchinsonii* chromosomal DNA with primers 1253inF and 1253inR, and inserted into the *Bam*HI/*Xba*I sites within the pLYL03 plasmid. To generate the pYT3198 plasmid, two DNA fragments corresponding to approximately 2 kb of *chu_3198* up- and downstream regions were amplified from *C. hutchinsonii* chromosomal DNA with the primers 3198upF/3198upR, and 3198downF/3198downR, respectively, and ligated into the pYT313 plasmid. For construction of the plasmids for complementation of the mutant strains, the *chu_1284* promoter sequence and the full-length CHU_3195 coding sequence were amplified from *C. hutchinsonii* genomic DNA with primers P1284F/P1284-3195R and P3195F/P3195R, respectively, and joined together by using overlap-extension PCR. The resultant DNA fragment was subsequently ligated into pCH03C to obtain the pCH3195 plasmid. The same constructive strategy was used to generate the plasmid pCH3199, which contains a DNA fragment placing the CHU_3199 coding sequence under the control of the *chu_1284* promoter. To construct the pCH3198 plasmid, the *ompA* promoter (Chen et al., 2007) and the CHU_3198 coding sequence were firstly amplified and fused together, and then inserted into pCH03C. All the primers used were listed in Table S1.

### Targeted insertion of *chu_3195, chu_3199, and chu_1253 and chu_3198* deletion

The plasmids pLYIN3195, pYT3198, pLYIN3199, and pLYIN1253 were individually transformed into *C. hutchinsonii* cells using electroporation as previously described (Zhu et al., 2013). After incubation on PY10 agar with erythromycin at 28°C for 7 days, resistant *C. hutchinsonii* colonies were picked, and transferred into liquid PY10 medium with erythromycin. For SacB-based deletion of *chu_3198*, the erythromycin-resistant cells were transferred to glucose-containing PY10 medium without any antibiotics. The propagated cells were harvested, diluted, and spread on PY10 agar plate with 0.4% glucose and 5% sucrose, followed by incubation at 28°C for 5–6 days. Single colonies were transferred into PY10 medium containing glucose and grown to exponential phase. The genomic DNA of the transformants was isolated using a Bacteria DNA kit (Sangon Biotech, Shanghai, China), and anchored PCR was performed to verify the correct integration events.

### Complementation of the *chu_3195, chu_3198*, and *chu_3199* mutants

The complementation plasmids pCH3195, pCH3198, and pCH3199 were individually introduced into the *chu_3195, chu_3198*, and *chu_3199* mutant strains using electroporation as described above. Cells were plated onto PY10 agar containing both erythromycin and chloramphenicol and cultured at 28°C for 5 days. The resistant colonies were then picked and cultured in liquid PY10 medium for plasmid extraction by using a plasmid mini kit (Omega Biotech, Doraville, USA). The isolated plasmids were then amplified via *E. coli* transformation and subjected to sequence verification.

### Growth analysis with different carbon sources

For growth analysis on different carbon sources, *C. hutchinsonii* cells pre-cultured in PY10 medium supplemented with 0.4% glucose were collected, washed twice, and transferred to fresh PY10 medium containing different carbon sources. When 0.4% glucose or 0.4% cellobiose was used as the carbon source in PY10 medium, *C. hutchinsonii* growth was monitored by measuring the optical density at 600 nm using a 96-well UV-visible spectrophotometer. When 0.2% crystalline cellulose (Avicel PH101, Sigma, St. Louis, USA) or 0.2% regenerated amorphous cellulose (RAC) was used as the sole carbon source, total cellular proteins reflecting the growth status were determined as previously described (Zhu et al., 2010). For growth assay on filter paper, cells grown to the exponential phase in liquid PY10 medium with 0.4% glucose were collected and washed with PY10 medium without any carbon sources. Equivalent amount of WT and mutant cells were then spotted on PY10 plate with filter paper on top of the agar (0.6%).

### Enzymatic assays

*C. hutchinsonii* cells cultured in PY10 medium with glucose to an OD_600_ of 0.3–0.4 were collected by centrifugation at 5,000 × g for 5 min, and then resuspended in 50 mM Piperazine-1,4-bis (2-ethanesulfonic acid) (PIPES) buffer at pH 6.8. Endoglucanase and β-glucosidase activities of the intact cells were determined essentially as previously described (Zhou et al., 2016), using sodium carboxymethyl cellulose (CMC-Na, Sigma-Aldrich, USA) and *p*-nitrophenyl β-D-glucopyranoside (*p*NPG, Sigma-Aldrich, USA) as the substrate, respectively. One unit of the CMCase activity or β-glucosidase activity was defined as the amount of the enzyme releasing 1 μmol of glucose or *p*NP per minute. For total protein concentration measurement, cells were washed once with 50 mM PIPES buffer at pH 6.8, resuspended in 0.2 M NaOH and being boiled for 20 min. Protein concentration was determined using the Bradford method (Bradford, 1976) and albumin from bovine serum (BSA) was used as standard. Specific activities was represented as U/mg of protein.

### SDS-PAGE analysis of extracellular supernatant proteins

*C. hutchinsonii* strains were grown in PY10 medium supplemented with 0.4% glucose at 28°C to mid-exponential phase with identical cell densities at OD_600_ between WT and mutant cells. The cultures were centrifuged at 5,100 × g for 20 min to collect cell pellet and cell-free supernatant, respectively. To precipitate proteins in the supernatant fraction, 56 ml of culture supernatant was supplemented with 14 ml of 50% trichloroacetic acid (TCA), incubated on ice for 12 h, and centrifuged at 13,000 × g for 30 min. The precipitate was finally dissolved in 90 μl of 1 × SDS-PAGE sample loading buffer after being washed twice with acetone. Samples with equal volumes were loaded for SDS-PAGE analysis. On the other hand, cell pellets were resuspended in 50 mM PIPES buffer at pH 6.8 after being washed with the same buffer, and subject to sonication. Cell lysates from WT and mutant strains with identical total protein amounts determined with a BCA protein assay kit (Pierce) were applied to SDS-PAGE analysis. Protein bands were visualized using Coomassie brilliant blue staining. Western blot analysis for CHU_0344 was performed as previously described (Wang et al., 2014).

### Isolation and identification of solubilized OM proteins adhered To cellulose

Preparation of the outer membranes (OMs), solubilization of the OM proteins (OMPs), and adhesion of OMPs to cellulose essentially followed the method described by Zhou et al. (2015) with some modifications. Specifically, cells were grown in PY10 medium supplemented with 0.4% glucose to mid-exponential phase and harvested at 5,000 × g for 10 min at 4°C, washed once with 50 mM PIPES buffer at pH 6.8 and resuspended in the same buffer supplemented with 0.5 M NaCl. The suspensions were vortexed thoroughly for 15 min and then centrifuged at 10,000 × g for 20 min to collect supernatant (S1) and sediment, respectively. Cells in sediments were subsequently resuspended with PIPES buffer containing 25% sucrose and vortexed once again for 10 min, followed by centrifugation at 10,000 × g for 20 min to collect supernatant fraction (S2) and sediment, respectively. The resultant sediment was subject to one repeated step with suspension buffer changed to distilled water and the resultant supernatant (S3) was collected. The mixed supernatant fractions (S1+S2+S3) were applied to ultracentrifugation at 100,000 × g for 1 h to sedimentate OMs. OM proteins were solubilized with 2% triton X-100 at 4°C for 12 h and ultracentrifuged at 100,000 × g for 30 min to remove insolubilized membrane debris. Equal amounts of solubilized OM proteins from WT and mutant strains were subject to SDS-PAGE analysis. To analyze the difference in cellulose-bound OM proteins between WT and mutant cells, ~0.5 mg solubilized OM proteins were mixed with 10 mg of autoclaved Avicel PH101 and rotated gently for 30 min at room temperature. The pelleted cellulose after centrifugation at 10,000 × g for 5 min was extensively washed twice with PIPES buffer. The OM proteins bound to cellulose were eluted with 1 × SDS-PAGE sample buffer, boiled for 10 min, and subject to SDS-PAGE. Protein bands were visualized using Coomassie brilliant blue staining. The missing or weakened protein bands in the profile of mutant strains compared to that of WT were cut and analyzed by matrix-assisted laser desorption/ionization time-of-flight (MALDI-TOF) mass spectrometry.

### Microscopic observation of colony spreading and individual cell motility

To examine *C. hutchinsonii* spreading, an aliquot of cells were transferred to an autoclaved glass slide that was covered with a thin layer of PY10 medium containing 0.6% agar, and incubated at 28°C for 2 days. The morphology of bacterial colony edges was observed by using phase contrast microscopy (Nikon Eclipse TE2000-S, Tokyo, Japan). Individual cell movement was examined essentially as described previously (Zhou et al., 2016). Briefly, the harvested cells were diluted in MMC buffer (10 mM MOPS, 4 mM MgSO_4_, 2 mM CaCl_2_, pH 7.6) to ~10^6^ cell/ml. Five microliters of the suspension was transferred onto glass slides, and then were overlaid with about 200 μl of 1% methylcellulose in MMC buffer and placed at room temperature for 1 h. The motility of *C. hutchinsonii* cells was monitored with inverted phase contrast microscope (Nikon Eclipse TE2000-S), and continuous images were recorded at 1 s intervals. The images were analyzed using the ImageJ software, and Microsoft videos (7 frames/s, wmv file) were exported resulting in a 7 × faster speed than real-time replay.

### Assay of cell adhesion to cellulose

Cell adhesion to Avicel was measured by using the turbidity-based method as previously described (Gong and Forsberg, 1989) with some modifications. Mid-exponential phase cells were harvested by centrifugation, washed twice with phosphate buffered saline buffer (137 mM NaCl, 2.7 mM KCl, 10 mM Na_2_HPO_4_, 2 mM KH_2_PO_4_, pH 7.4), and resuspended in the same buffer to an optical density at 600 nm of 1.0 (A_0_). A 3.5-ml cell suspension was thoroughly mixed with 0.5 ml 10% (w/v) autoclaved Avicel. The mixture was left to stand at room temperature for 40 min with gentle rotation. The cell density of the resultant supernatant after sedimentation was again determined by measuring the OD_600nm_(A_1_). Calculation of the percentage of cellulose-adhered cells was carried out using the following equation:

$$\begin{array}{l} A_{2}=\frac{A_{0}-A_{1}}{A_{0}}\times 100\% \end{array}$$

*A*_0_: OD_600nm_ value before adsorption; *A*_1_: OD_600nm_ value after adsorption; *A*_2_: percentage of adhered cells.

To further compare the adsorbing affinity of WT and mutant cells to cellulose during incubation, cellulose with adhered WT or mutant cells obtained above were pelleted, resuspended in the same volume buffer, and was incubated at room temperature with gentle rotation. The cell density of the supernatant after sedimentation (A_t_) was measured at the indicated time point, and the percentage of cells retaining on cellulose was calculated with the following equation:

$$\begin{array}{l} A_{3}=\frac{A_{0}-A_{1}-A_{t}}{A_{0}-A_{1}}\times 100\% \end{array}$$

*A*_*t*_: OD_600 nm_ value after incubation for different time periods; *A*_3_: percentage of cells retaining to cellulose.

### Electron microscopic analysis

Cells for scanning electron microscopic analysis were prepared as described by Xie with some minor modifications (Xie et al., 2007). Briefly, cells grown to the exponential phase on glucose were spotted onto filter paper on top of PY10 agar and incubated at 28°C for 40 min. The filter paper was then transferred to a tube, suspended in phosphate buffered saline buffer, and incubated at room temperature with low-speed shaking for the indicated time period. The cells adhered to filter paper was fixed with 2.5% glutaraldehyde in 100 mM PBS buffer (pH 7.2) at 4°C for 12 h, followed by washing with 100 mM PBS buffer, and dehydrated with ethanol. Samples were mounted, sputter coated with 60% gold and 40% palladium, and finally viewed with a JEOL JSM-7600F scanning electron microscope.

### Statistical analysis

Statistical analysis was performed using the student's *t*-test analysis. At least three biological replicates were performed for each analysis and the results and errors are the mean and SD, respectively, from three to five replicates.

### Search of T2SS component and sequence analyses

To search for the presence of homologous T2SS genes in *C. hutchinsonii* genome, the well-characterized T2SS protein sequences from several *Proteobacteria* members and the InterPro and Pfam numbers for T2S-D (IPR004846, PF00263), T2S-E (IPR001482, PF00437), T2S-F (IPR018076, PF00482), T2S-G (IPR013545, PF08334), T2S-L (IPR024230, PF05134), T2S-M (IPR007690, PF04612), and T2S-C (IPR024961, PF11356) were used as queries. Multiple sequence alignments were performed using Clustal W (Larkin et al., 2007). Pfam domain identification and secondary structural analysis was carried out using the Pfam 31.0 tool (http://pfam.xfam.org/) and the PSIPRED Server (http://bioinf.cs.ucl.ac.uk/psipred/), respectively (Jones, 1999; Finn et al., 2016). HHpred analyses of protein sequences were undertaken using the server available online (https://toolkit.tuebingen.mpg.de; Soding et al., 2005).

## Results

### Identification of a gene cluster encoding a putative type II secretion system with a distinct organization in *C. hutchinsonii* genome

**Figure 1:**
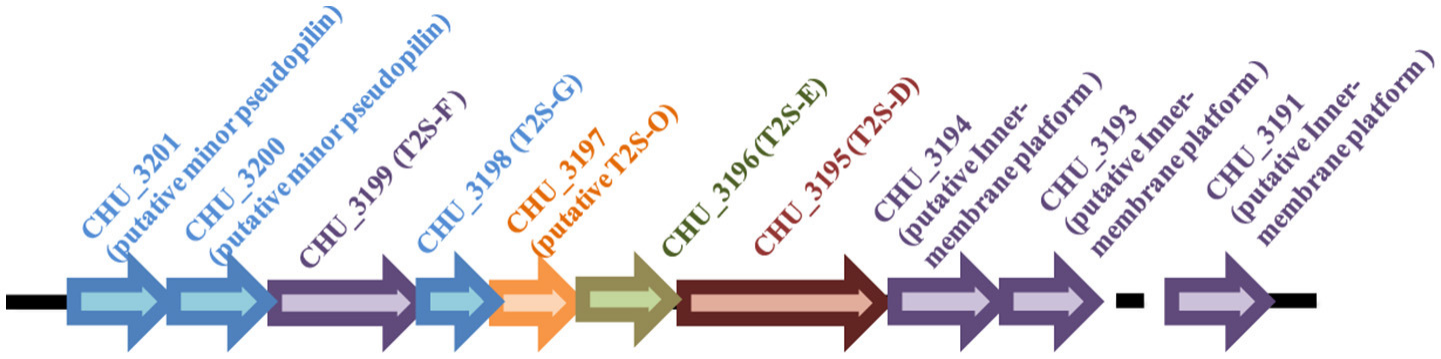
Schematic illustration of the organization of the *t2s* gene loci in *C. hutchinsonii* genome.

Gram-negative bacteria use protein secretion systems to transport proteins across the outer membrane (Desvaux et al., 2009; Chagnot et al., 2013). Among others, the conserved type II secretion systems (T2SSs) transport a wide variety of folded proteins from the periplasm into the extracellular environment or the outer membrane surface (Korotkov et al., 2012; Rondelet and Condemine, 2013; Cianciotto and White, 2017). Due to the lack of systematic characterization of *Bacteroidetes* T2SS genes, the well-characterized T2SS protein sequences from several *Proteobacteria* members were used as queries to search for the presence of homologous T2SS genes in *C. hutchinsonii* genome. Genes encoding the four major homologous components of T2SS were localized via analysis on KEGG website. Whereas *chu_3195* and *chu_3199* encode orthologs of the major outer-membrane protein T2S-D, often referred to as secretin, and the inner-membrane protein T2S-F, respectively, *chu_3196* and *chu_3198* encode the secretion ATPase T2S-E and the major pesudopilin T2S-G, respectively. The four genes are organized into a cluster together with several upstream or downstream genes encoding putative hypothetical proteins via BLAST analysis (*chu_3197, chu_3200*-*chu_3202, chu_3194, chu_3193*, and *chu_3191*; Figure 1). Further structural analyses revealed that, while CHU_3197 was predicted to possess a Pfam domain Peptidase_A24 and several transmembrane helices, which is commonly observed in typical prepilin peptidase T2S-O, CHU_3200, and CHU_3201 were found to share sequence homology with well-characterized minor pseudopilins at their N-terminal hydrophobic alpha-helix comprising ~20 residues (Bleves et al., 1998; Korotkov and Hol, 2008; Figure S1). Moreover, analyses of CHU_3194, CHU_3193, and CHU_3191 using the HHpred server for remote protein homology detection retrieved several hits to PilN/M/O/P, the type 4 secretion system components which are evolutionally related with T2S-L/M/C (Korotkov and Hol, 2008), suggesting that they are possible candidates for the orthologs of the inner-membrane spanning proteins T2S-L/M/C, although no Pfam domain or significant sequence similarity are detected for these sequences. The putative T2SS gene clusters were also found in four other *Bacteroidetes* members with sequenced genomes (Table 2). In addition to the remarkably variable sequences of the minor pseudopilins and the inner-membrane platform constituents, the gene organization of the identified *Bacteroidetes t2s* clusters was also found to be obviously distinct from the well characterized prototype in *Proteobacteria* (Figure S2). Moreover, unlike the typical T2SS genes found in many *Proteobacteria* bacteria (e.g., *E. coli, K. oxytoca*, and *Vibrio cholerae*), which are clustered in one locus in the chromosome and organized into an operon under the control of one common promoter, all the putative T2SS genes in *C. hutchisonii* may not be organized in an operon as a whole, as evidenced by the observation that only the sequences spanning the ends of *chu_3200* and *chu_3201, chu_3197* and *chu_3198*, respectively, could be amplified using reverse transcriptional PCR (Figure S3).

**Table 2:** Distribution of T2SS components in *E. coli, C. hutchinsonii, and four other Bacteroidetes* members.

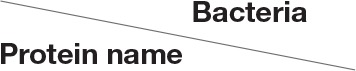		***E. coli*^*a*^**	***C. hut*^*a*^**	***F. joh*^*a*^**	***P. hep*^*a*^**	***O. spl*^*a*^**	***F. psy*^*a*^**
Inner-membrane platform protein	T2S-F T2S-C T2S-L T2S-M	B3327 25%^b^ B3324 B3333 B3334	CHU_3199 CHU_3194 CHU_3193 CHU_3191	Fjoh_0614 38%^b^ Fjoh_0619 Fjoh_0620 Fjoh_0622	Phep_1112 Phep_1104 Phep_1105 Phep_1107	Odosp_1507 Odosp_1525 Odosp_1524 Odosp_1522	
Outer-membrane secretin	T2S-D	B3325	CHU_3195	Fjoh_0618 38%^b^	Phep_1103 45%^b^	Odosp_1526 29%^b^	FP2299 46%^b^
Secretion ATPase	T2S-E	B3326 42%^b^	CHU_3196	Fjoh_0617 52%^b^	Phep_1102 59%^b^	Odosp_1527 49%^b^	FP2300 36%^b^
Major pseudopilin	T2S-G	B3328	CHU_3198	Fjoh_0615 50%^b^	Phep_1100 70%^b^	Odosp_1529 62.5%^b^	FP2302 53%^b^
Minor pseudopilin	T2S-H T2S-I T2S-J T2S-K	B3329 B3330 B3331 B3332	CHU_3200 CHU_3201	Fjoh_0611 Fjoh_0612 Fjoh_0613	Phep_1111 Phep_1110		
Prepilin peptidase	T2S-O	B3335	CHU_3197	Fjoh_0616	Phep_11031	Odosp_1528	FP2301

a *E. coli, Escherichia coli (Hayashi et al., 2006); C. hut, Cytophaga hutchinsonii ATCC 33406 (Xie et al., 2007); F. joh, Flavobacterium johnsoniae (McBride et al., 2009); F. psy, Flavobacterium psychrophilum (Han et al., 2009); P. hep, Pedobacter heparinus (Duchaud et al., 2007); O. spl, Odoribacter splanchnicus (Goker et al., 2011)*.

b *The protein sequence identities (>25%) between C. hutchinsonii T2S and its orthologs*.

### Mutants lacking the individual orthologous *t2s* genes are defective in cellulose utilization

**Figure 2:**
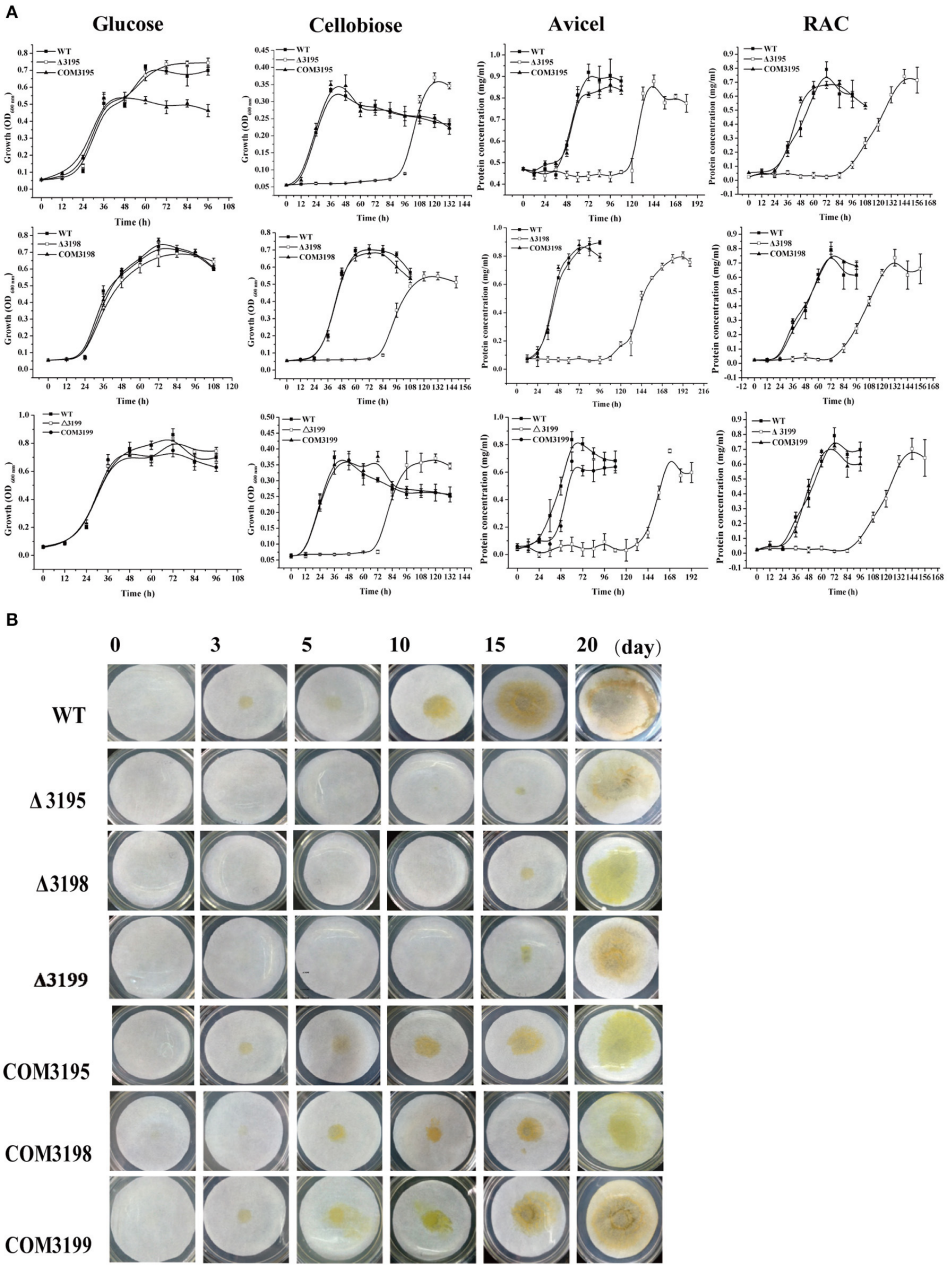
Growth analyses of *C. hutchinsonii* WT, *t2s* mutant strains and the individual complemented strains in liquid PY10 medium containing different carbon sources as indicated **(A)** and on solid plate with filter paper on top of PY10 agar **(B)**. The growth assay was performed in duplicate (with the same results) and one representative result is shown.

To test whether the identified T2SS gene cluster is involved in *C. hutchinsonii* cellulose utilization, mutants with targeted insertional inactivation of *chu_3195, chu_3199*, and a mutant lacking *chu_3198* were constructed (Figure S4). Whereas growth of the three mutants was largely comparable to that of WT on glucose, a significantly long lag in growth was observed on cellobiose with the growth starting only until after 72 h-cultivation, although the final biomass yield was comparable to that of WT strain (Figure 2). The mutant growth was also delayed until after about 100 h when cultured on RAC (Figure 2). When analyzed for the ability to digest filter paper cellulose and Avicel, all the three mutants exhibited roughly the same phenotypes displaying a significantly compromised growth with detectable growth only until after 108 h-cultivation on Avicel and 10 d-cultivation on filter paper. The deficient growth was largely restored when the mutants were individually complemented with a plasmid expressing the respective *t2s* genes, indicating that the deficiency in cellulose utilization by these mutants was specifically caused by the targeted disruption of the respective gene locus.

**Figure 3:**
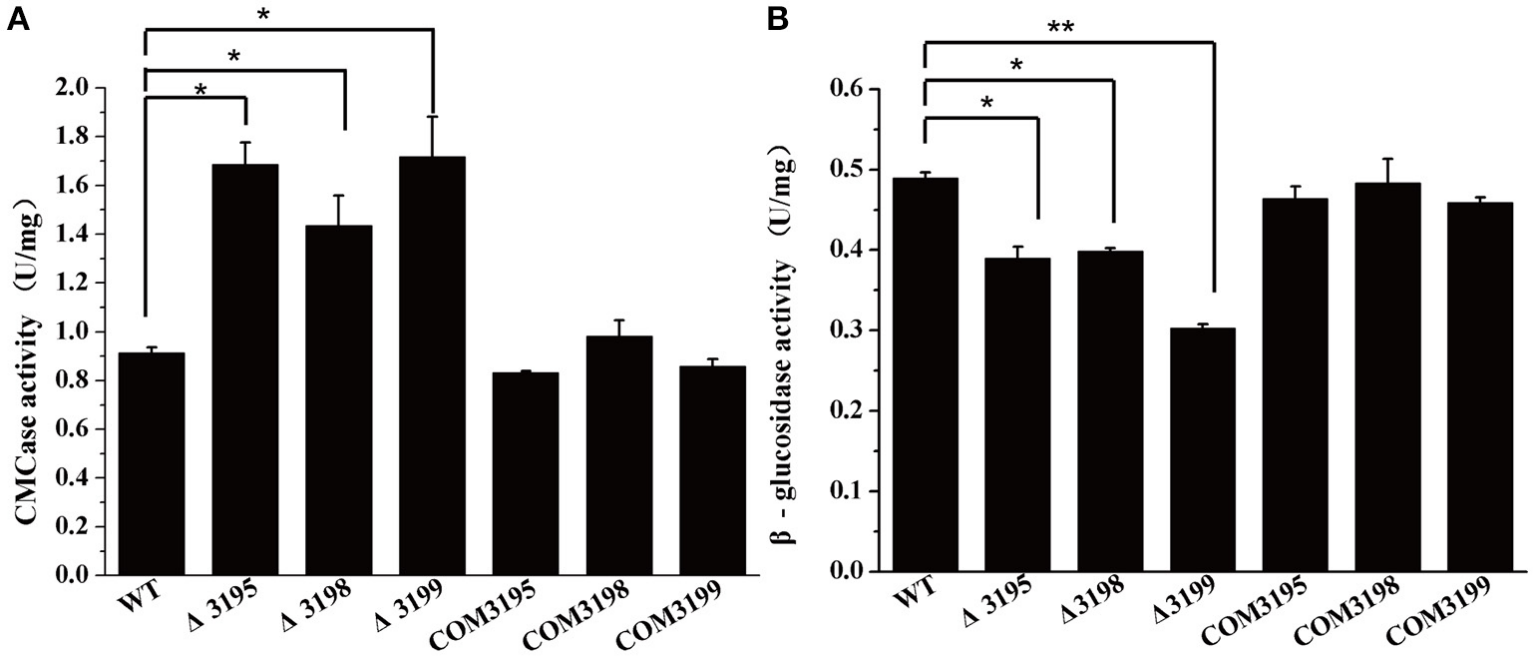
Endoglucanase **(A)** and β-glucosidase **(B)** activity of the intact cells of WT, *t2s* mutant strains, and *t2s* complemented strains. Statistical analysis was performed using the student's *t*-test analysis. Significant differences (*T*-test ^*^P < 0.05, ^**^P < 0.01) in the enzymatic activities were detected between WT and *t2s* mutant strains. Values in this figure are the mean of three to five biological replicates. Error bars are the SD from these replicates.

To test whether the deficiency in cellulose utilization displayed by *t2s* mutants resulted from the altered hydrolytic activities associated with *C. hutchinsonii*, the specific carboxymethylcellulase (CMCase) and β-glucosidase activities of intact WT and mutant cells were determined. As shown in Figure 3A, whereas the CMCase activity of the individual mutant was two- to three-fold higher than that of WT, the β-glucosidase activity was about 40% lower than that of WT, which may account for the growth delay on cellobiose (Figure 3B).

### T*2s* mutants were deficient in cellulose-bound outer membrane proteins

**Figure 4:**
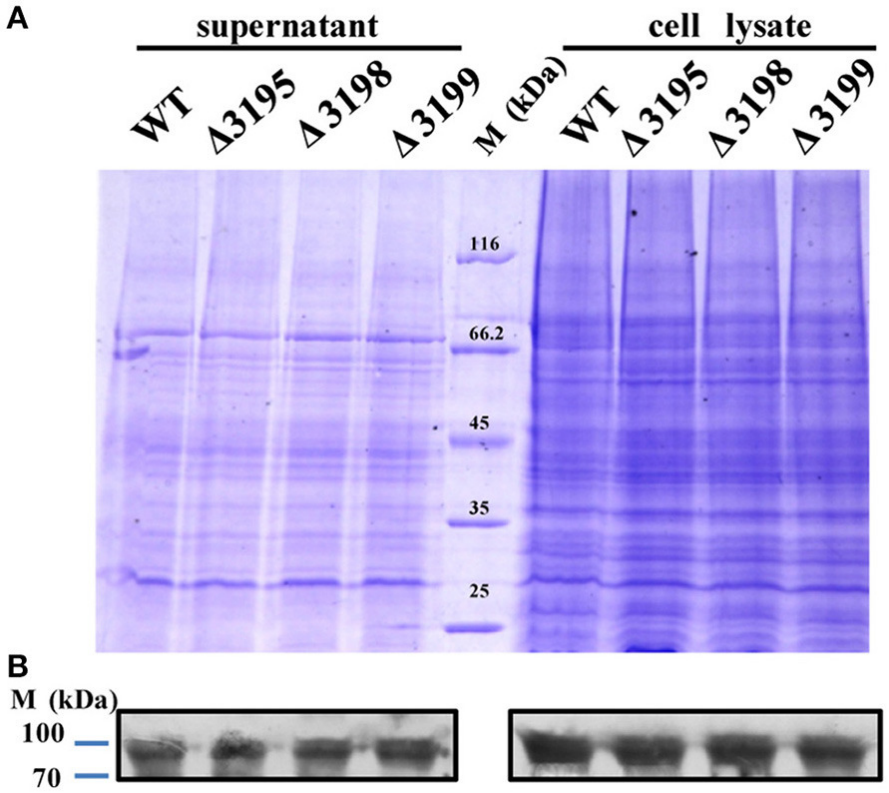
SDS-PAGE analyses of the extracellular supernatant and cell lysate from WT and *t2s* mutant strains **(A)** and western blot detection for the extracellular or intracellular CHU_0344 protein using the antibody against CHU_0344 **(B)**. Loading samples were normalized by calibration of cell biomass or total proteins. Proteins were stained with Coomassie brilliant blue. The signals of CHU_0344 with almost the same intensity from different cell lysates verified an equal protein loading between them.

**Figure 5:**
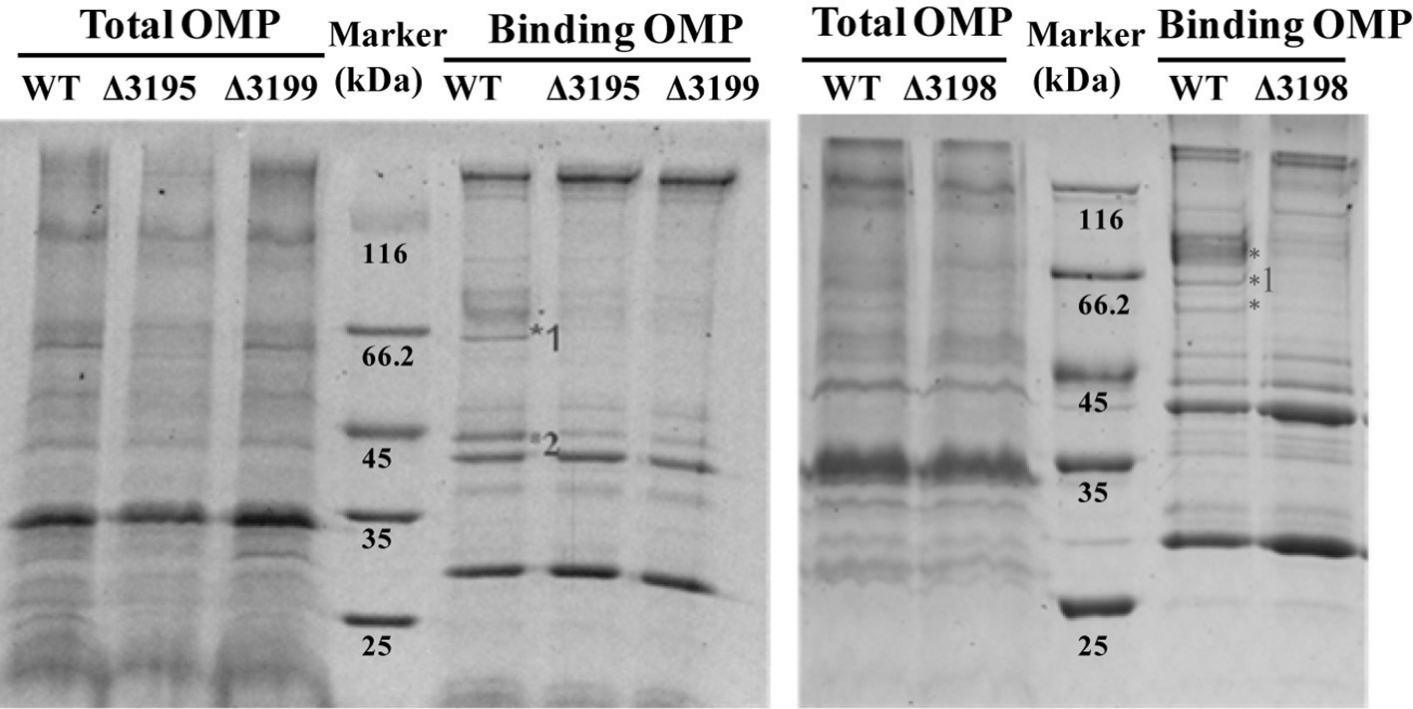
SDS-PAGE analyses of the cellulose-bound outer membrane proteins (OMPs) isolated from WT and the *t2s* mutant strains cultured from PY10 medium supplemented with 0.4% glucose. Total OMPs amounts were calibrated for equal protein loading. Proteins were stained with Coomassie brilliant blue, and the figure color was adjusted as black and white for clear visualization. The bands missing or weakened in the profile from the mutant strains relative to that from WT were indicated by asterisk. The bands denoted with numbers were successfully identified to be CHU_1277 and CHU_1253, respectively by mass spectral analysis.

**Figure 6:**
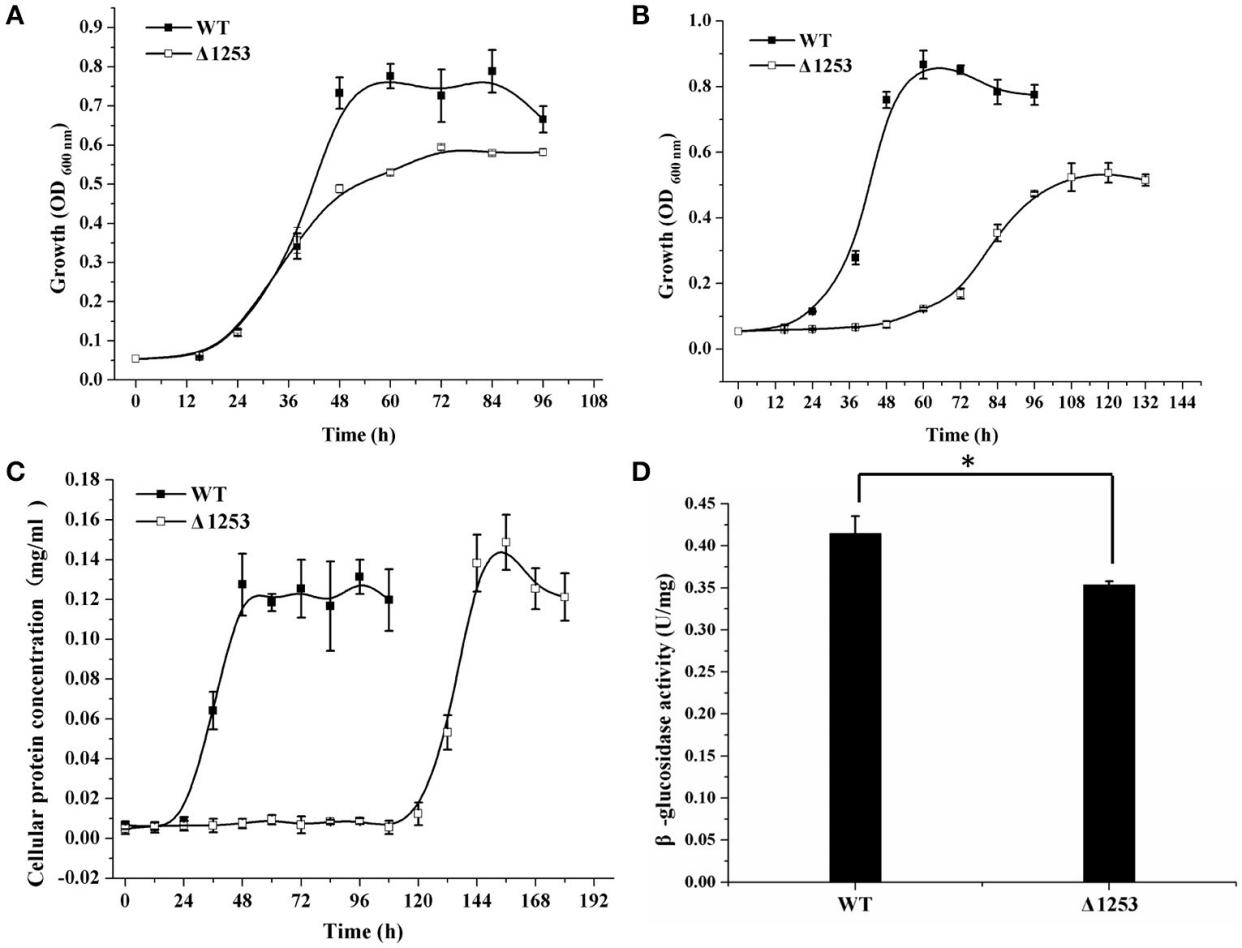
Analyses of growth and β-glucosidase activity of the *chu_1253*-inactivated mutant and WT strains. **(A)** Cell growth on glucose, **(B)** Cell growth on cellobiose, **(C)** Cell growth on Avicel, **(D)** β-glucosidase activity of the intact cells of WT and the mutant strains. Statistical analysis was performed using the student's *t*-test analysis. Significant differences (*T*-test ^*^P < 0.05) in the enzymatic activities were detected. Values in this figure are the mean of three to five biological replicates. Error bars are the SD from these replicates.

Considering that a vast majority of T2SS substrates are secreted to extracellular milieu, we first focused on the effect of the T2SS absence on extracellular soluble proteins produced by *C. hutchinsonii*. As shown in Figure 4A, hardly any difference was observed for the profiles of the resolved proteins when cell-free spent culture medium from wild-type and other T2SS mutant cells was analyzed by SDS-PAGE. Accordingly, production of one of the prominent extracellular proteins, CHU_0344, which contains PKD and Ig-like domains that may be involved in cellulose substrate adhesion (Xu et al., 2012), was not affected either in the T2SS mutants by western blot analysis (Figure 4B). To identify the differentially expressed outer membrane (OM) proteins potentially resulting from the T2SS absence, OM proteins extracted from WT and the individual *t2s* mutant were bound to cellulose and were resolved by SDS-PAGE (Figure 5 and Figure S5). Several protein bands that were missing or weakened in the profile from the mutant cells compared to that from WT cells, were subject to mass spectroscopic analysis for protein identification (Table S2). The two successfully identified bands (denoted with numbers 1 and 2) corresponded to CHU_1277 and CHU_1253 respectively, of which CHU_1277 was previously shown to be critical for *C. hutchinsonii* cellulose utilization via participation in cellooligosaccharide assimilation (Ji et al., 2014). Further inserted inactivation of *chu_1253* revealed that the absence of CHU_1253 also compromised *C. hutchinsonii* cellulose utilization with decreased β-glucosidase activity (Figure 6).

### T*2s* mutants retained moving ability

**Figure 7:**
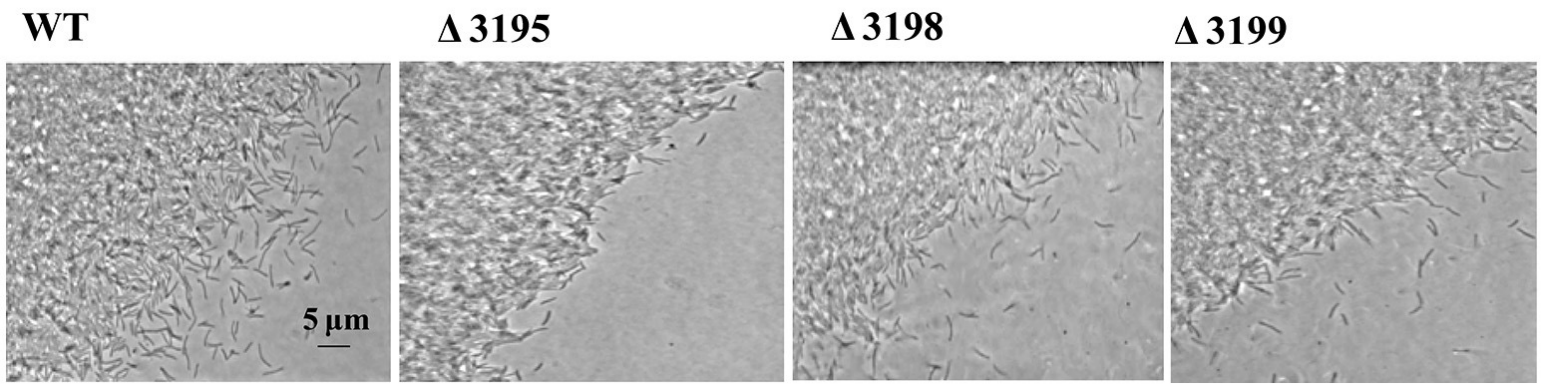
Phase contrast microscopic analysis of the colony periphery of WT and *t2s* mutant cells on 0.6% agar surface.

Gliding along cellulose fibers has been proposed to facilitate the cellulose digestion by *C. hutchinsonii* (Stanier, 1942; Xie et al., 2007). To determine whether the absence of T2SS components affect cell movement, WT and *t2s* mutant cells were examined for colony spreading abilities. As shown in Figure 7, the behavior of the mutant cells at the edge of the colony was observed to be largely indistinguishable from that of WT when cultivated on 0.6% agar except the *chu_3195* mutant, whose colony boundary was relatively more obvious and compact. Cells were further examined for moving ability in viscous methylcellulose medium to minimize the random Brownian motion. As shown in Movies S1–S4 in the Supplementary Material, there was no apparent difference in the rapid motility of individual mutant cells compared with that of WT cells. These results imply that the putative T2SS may not be involved in the appropriate processing of cell surface proteins that are required for motility.

### T*2s* mutant strains were compromised in adhesion to cellulose

**Figure 8:**
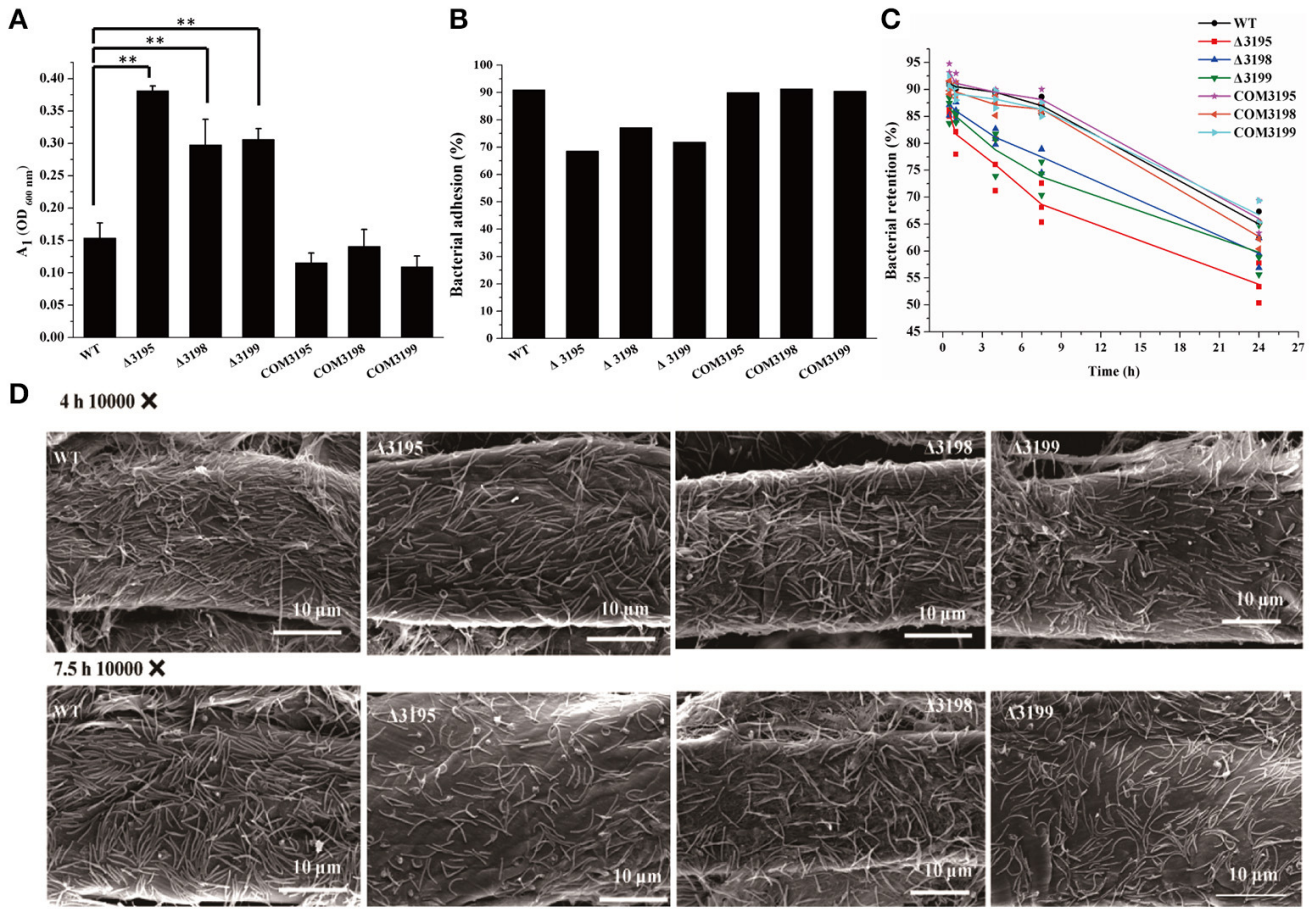
Analyses of WT and *t2s* mutant cell adhesion to cellulose. **(A)** The cell density (A_1_, OD_600 nm_) of the resultant supernatant after bacterial adhesion to cellulose at room temperature for 40 min with gentle rotation. The cell density at 600 nm (A_0_) before adhesion was adjusted to 1.0 for all the strains. Statistical analysis was performed using the student's *t*-test analysis. Significant differences (*T*-test ^**^P < 0.01) were detected between WT and *t2s* mutant strains. **(B)** The percentage of cells adhered to Avicel cellulose after gentle rotation at room temperature for 40 min as indicated in **(A). (C)** The percentage of WT and mutant cells retained on cellulose after incubation for the indicated time periods. The respective percentage of the pre-adhered WT and *t2s* mutant cells as described in panel B was set to 100%. Three independent results for each strain were shown. The equations used for calculation of the cell percentage were described in Section Materials and Methods. **(D)** Scanning electron microscopic analyses of WT and mutant cells adhered to filter paper after incubation for the indicated time periods. Panels from left to right: WT, Δ3195, Δ3198, and Δ3199.

Adhesion of *C. hutchinsonii* cells to insoluble cellulose is thought to be required for efficient cellulose degradation though genomic analysis failed to detect any obvious CBPs (Larkin, 1989; Xie et al., 2007). Whereas each of the T2SS mutants retained the ability to adhere to cellulose, a significant decrease in the adhering capability was observed compared with that of the WT strain (Figures 8A,B). The percentage of WT cells, and of *chu_3195, chu_3198*, and *chu_3199* mutant cells that attached to Avicel was 92, 67, 75, 70%, respectively. To verify the defect in cellulose adhesion as displayed by the mutants, cells attached to Avicel were resuspended and incubated with gentle rotation for the indicatedtime periods to determine the percentageof cells remaining attached. WhereasWT cells maintained a tightadhesion to Avicel even after an incubation of 7.5 h, there was a significant decreasein the percentage of adhered mutantcells to Avicel (Figure 8C). The difference between theadhering ability of WT and mutantcells to filter paper was further examined by scanning electron microscopic analysis after a similar treatment as above. Whereas WT cells were densely populated but regularly aligned in a head-to-tail mode on the surface of filter paper, the mutant cells were sparsely distributed in a relatively disordered way (Figure 8D and Figure S6). The decreased adhesion of the T2SS mutants to cellulose suggests that the predicted *C. hutchinsonii* T2SS may be involved in the secretion of proteins mediating cell attachment to cellulose. Additional studies are needed to determine the identity and functions of these proteins, and to characterize the mechanism by which the identified T2SS secrets them.

## Discussion

The cellulolytic bacterium *C. hutchinsonii* is considered to degrade cellulose in a substrate contact-dependent manner (Stanier, 1942; Larkin, 1989), and specific proteins are speculated to be secreted to extracellular milieu or outer membrane to participate in cellulose digestion. The type II secretion system (T2SS) comprising 12–15 proteins is devoted to translocate a wide variety of proteins from the periplasm and into extracellular milieu or outer membrane surface (DiChristina et al., 2002; Le Blastier et al., 2010; Baldi et al., 2012; Cianciotto and White, 2017). It has been extensively studied in plant and animal pathogenic bacteria, but rarely studied in nonpathogenic bacteria. The T2SS genes are commonly observed to be clustered in one or two loci, whereas some are dispersed in more than two loci scattered through the chromosome, especially those from *Acinetobacter baumannii* and *Legionella pneumophila*, which are dispersed in up to five different loci (Rossier et al., 2004; Harding et al., 2016). In this study, we identified a gene cluster encoding a putative T2SS involved in cellulose assimilation by the cellulolytic bacterium *C. hutchinsonii*. Whereas BLAST analysis with well-characterized T2SS sequences from *Proteobacteria* members as queries only retrieved orthologs of *t2s-D* (*chu_3195*), *t2s-E* (*chu_3196*), *t2s-G* (*chu_3198*), and *t2s-F* (*chu_3199*), further systematic analyses using Pfam and HHpred tools identified additional *t2s* genes with highly variable sequences, including the prepilin peptidase T2S-O-encoding gene *chu_3197*, the minor pseudopilins-encoding genes *chu_3200* and *chu_3201* as well as the inner-membrane platform constitute-encoding genes *chu_3194, chu_3193*, and *chu_3191*, which were all previously annotated as hypothetical proteins with unknown functions. In addition to the divergent sequences, the organization of the identified T2SS gene clusters from *C. hutchinsonii* and four other *Bacteroidetes* members were also found to be obviously distinct from the well characterized prototype in many *Proteobacteria* members such as *E. coli, K. oxytoca*, and *V. cholerae*. Of note, minor pseudopilins are yet to be found in *Flavobacterium psychrophilum* and *Odoribacter splanchnicus*, suggesting a significantly evolutionary divergence of *t2s* genes even in the phylum. Moreover, unlike the typical T2SS genes which are distributed in one locus and organized into an operon under the control of one common promoter, all the putative *C. hutchisonii* T2SS genes, though clustered in one locus, are most likely not organized in an operon as a whole. However, conclusion cannot be drawn at present that the above identified putative T2SS gene products are indeed functional counterparts of the well characterized T2SS given the scarce information from the closely related *Bacteroidetes* members. Possibility also exists that other possible candidates with more divergence from the typical T2SS components are present somewhere in the genome.

Whereas orthologues of T2S-D, T2S-F, and T2S-G in *C. hutchinsonii* (CHU_3195, CHU_3198, and CHU_3199) play an important role in cellulose degradation, as indicated by the significantly retarded growth of the individual mutant on cellobiose, Avicel, RAC, and filter paper, they do not have a noticeable effect on cell motility. Previous studies demonstrated that two orthologous components (SprP and PorU) of the recently identified secretion system T9SS are required for both *C. hutchinsonii* cellulose digestion and gliding motility (Wang et al., 2014; Zhu and McBride, 2014). This differential roles of T2SS and T9SS orthologues in *C. hutchinsonii* cellulose utilization and gliding motility are probably correlated with their different involvement in maintaining the proper function of specific proteins, which has been largely unknown so far. We also noted that the *chu_3195* (*t2s-D*) mutant displayed a bit more apparent defect in total OM proteins, colony spreading and bacterial adherence to cellulose than the other two mutants although its viability is not noticeably compromised. Moreover, no detectable difference in cell morphology between the *chu_3195* mutant and WT strains could be observed by using scanning or transmission electron microscopic analyses (Figure S7). It is not clear at present whether CHU_3195 fulfills additional functions besides being a secretin in T2SS. Considering its potential localization on the outer membrane, one can speculate that the absence of CHU_3195 might exert some other effects on the presentation of outer membrane or cell-surface-adhesive proteins, making this mutant more sensitive to extracellular stresses, and thus exhibit a more apparent defect than the other two mutants.

To explore the potential proteins secreted by T2SS in *C. hutchinsonii* which may be responsible for the deficiency in cellulose degradation in the T2SS mutants, we examined the effect of the individual absence of the three T2SS components on the extracellular proteins as well as outer membrane proteins bound to cellulose from WT and mutant cells. Whereas, the extracellular protein profiles were hardly affected in the mutants, outer membrane proteins bound to cellulose revealed apparently different profiles. Notably, CHU_1277 that is missing in the mutants encodes a putative outer membrane protein, whose absence results in a decline in the cell-associated β-glucosidase activity and almost completely abolishes cellulose degradation by *C. hutchinsonii* (Ji et al., 2014). The observed reduction in β-glucosidase activities in the *t2s* mutants may thus result from their inappropriate presentation due to the malfunction of *chu_1277* or *chu_1276* gene products, both of which have been reported to be important in maintaining the cell-associated β-glucosidase activities that are tightly coupled with cellulose utilization (Ji et al., 2014; Zhou et al., 2016). This altered organization or presentation of the relevant enzymes on the cell surface as seen with the changes in outer membrane protein profiles in *t2s* mutants may also account for the observed increased endoglucanase activity. Similarly, the targeted inactivation of *chu_1253*, the other compromised outer membrane protein, led to a defect in cellulose utilization together with reduced cell-associated β-glucosidase activity. Given that CHU_1277 and CHU_1253 are outer membrane porins, which are not likely to be the direct substrates of T2SS, we speculate that the deficiency in cellulose utilization as demonstrated by the *t2s* mutants was correlated with the compromised organization or presentation of the outer membrane proteins including relevant enzymes and lipoproteins, which has also been reported to occur in other bacteria (Howard et al., 1993; Vignon et al., 2003; Sikora et al., 2007). Further corroborating this assumption is the observation that the absence of T2SS components exerted a significant effect on the cell adhesion to cellulose, which is a prerequisite for *C. hutchinsonii* cellulose degradation and is believed to involve a set of cell surface proteins to facilitate cell-cellulose interaction. Whereas, the T2SS mutant cells retained the ability to adsorb to cellulose, the adhering capability was significantly decreased compared to the WT cells. Identification and characterization of these compromised cell surface-adhesins as well as outer membrane proteins whose appropriate presentation is mediated by T2SS will definitely help our understanding the unique cellulolytic mechanism deployed by *C. hutchinsonii*.

## Author contributions

XW and QH performed the experiments. WL, WZ, and GC performed data analysis. WL designed the project. XW, WZ, and WL wrote the manuscript.

### Conflict of interest statement

The authors declare that the research was conducted in the absence of any commercial or financial relationships that could be construed as a potential conflict of interest.

## References

[B1] Baldi D. L. Higginson E. E. Hocking D. M. Praszkier J. Cavaliere R. James C. E.. (2012). The type II secretion system and its ubiquitous lipoprotein substrate, SslE, are required for biofilm formation and virulence of enteropathogenic *Escherichia coli*. Infect. Immun. 80, 2042–2052. 10.1128/IAI.06160-11 22451516 PMC3370571

[B2] Bleves S. Voulhoux R. Michel G. Lazdunski A. Tommassen J. Filloux A. (1998). The secretion apparatus of *Pseudomonas aeruginosa*: identification of a fifth pseudopilin, XcpX (GspK family). Mol. Microbiol. 27, 31–40. 10.1046/j.1365-2958.1998.00653.x 9466253

[B3] Bradford M. M. (1976). A rapid and sensitive method for the quantitation of microgram quantities of protein utilizing the principle of protein-dye binding. Anal. Biochem. 72, 248–254. 10.1016/0003-2697(76)90527-3 942051

[B4] Braun T. F. Khubbar M. K. Saffarini D. A. McBride M. J. (2005). Flavobacterium johnsoniae gliding motility genes identified by mariner mutagenesis. J. Bacteriol. 187, 6943–6952. 10.1128/JB.187.20.6943-6952.2005 16199564 PMC1251627

[B5] Chagnot C. Zorgani M. A. Astruc T. Desvaux M. (2013). Proteinaceous determinants of surface colonization in bacteria: bacterial adhesion and biofilm formation from a protein secretion perspective. Front. Microbiol. 4:303. 10.3389/fmicb.2013.00303 24133488 PMC3796261

[B6] Chen S. Bagdasarian M. Kaufman M. G. Bates A. K. Walker E. D. (2007). Mutational analysis of the ompA promoter from *Flavobacterium johnsoniae*. J. Bacteriol. 189, 5108–5118. 10.1128/JB.00401-07 17483221 PMC1951883

[B7] Cianciotto N. P. (2005). Type II secretion: a protein secretion system for all seasons. Trends Microbiol. 13, 581–588. 10.1016/j.tim.2005.09.005 16216510

[B8] Cianciotto N. P. White R. C. (2017). Expanding role of type II secretion in bacterial pathogenesis and beyond. Infect. Immun. 85:e00014–17. 10.1128/IAI.00014-17 28264910 PMC5400843

[B9] Desvaux M. Hebraud M. Talon R. Henderson I. R. (2009). Secretion and subcellular localizations of bacterial proteins: a semantic awareness issue. Trends Microbiol. 17, 139–145. 10.1016/j.tim.2009.01.004 19299134

[B10] Desvaux M. Parham N. J. Scott-Tucker A. Henderson I. R. (2004). The general secretory pathway: a general misnomer? Trends Microbiol. 12, 306–309. 10.1016/j.tim.2004.05.002 15223057

[B11] DiChristina T. J. Moore C. M. Haller C. A. (2002). Dissimilatory Fe(III) and Mn(IV) reduction by *Shewanella putrefaciens* requires ferE, a homolog of the pulE (gspE) type II protein secretion gene. J. Bacteriol. 184, 142–151. 10.1128/JB.184.1.142-151.2002 11741854 PMC134750

[B12] Duchaud E. Boussaha M. Loux V. Bernardet J. F. Michel C. Kerouault B.. (2007). Complete genome sequence of the fish pathogen *Flavobacterium psychrophilum*. Nat. Biotechnol. 25, 763–769. 10.1038/nbt1313 17592475

[B13] Finn R. D. Coggill P. Eberhardt R. Y. Eddy S. R. Mistry J. Mitchell A. L.. (2016). The Pfam protein families database: towards a more sustainable future. Nucleic Acids Res. 44, D279–285. 10.1093/nar/gkv1344 26673716 PMC4702930

[B14] Goker M. Gronow S. Zeytun A. Nolan M. Lucas S. Lapidus A.. (2011). Complete genome sequence of Odoribacter splanchnicus type strain (1651/6). Stand. Genomic Sci. 4, 200–209. 10.4056/sigs.1714269 21677857 PMC3111987

[B15] Gong J. Forsberg C. W. (1989). Factors affecting adhesion of *Fibrobacter succinogenes* subsp. succinogenes S85 and adherence-defective mutants to cellulose. Appl. Environ. Microbiol. 55, 3039–3044. 2619302 10.1128/aem.55.12.3039-3044.1989 PMC203220

[B16] Green E. R. Mecsas J. (2016). Bacterial secretion systems: an overview. Microbiol. Spectr. 4:VMBF-0012-2015. 10.1128/microbiolspec.VMBF-0012-2015 26999395 PMC4804464

[B17] Han C. Spring S. Lapidus A. Del Rio T. G. Tice H. Copeland A.. (2009). Complete genome sequence of Pedobacter heparinus type strain (HIM 762-3). Stand. Genomic Sci. 1, 54–62. 10.4056/sigs.22138 21304637 PMC3035210

[B18] Harding C. M. Kinsella R. L. Palmer L. D. Skaar E. P. Feldman M. F. (2016). Medically relevant acinetobacter species require a type II secretion system and specific membrane-associated chaperones for the export of multiple substrates and full virulence. PLoS Pathog. 12:e1005391. 10.1371/journal.ppat.1005391 26764912 PMC4713064

[B19] Hayashi K. Morooka N. Yamamoto Y. Fujita K. Isono K. Choi S.. (2006). Highly accurate genome sequences of *Escherichia coli* K-12 strains MG1655 and W3110. Mol. Syst. Biol. 2, 2006.0007. 10.1038/msb4100049 16738553 PMC1681481

[B20] Howard S. P. Critch J. Bedi A. (1993). Isolation and analysis of eight exe genes and their involvement in extracellular protein secretion and outer membrane assembly in *Aeromonas hydrophila*. J. Bacteriol. 175, 6695–6703. 10.1128/jb.175.20.6695-6703.1993 8407845 PMC206782

[B21] Ji X. Wang Y. Zhang C. Bai X. Zhang W. Lu X. (2014). Novel outer membrane protein involved in cellulose and cellooligosaccharide degradation by *Cytophaga hutchinsonii*. Appl. Environ. Microbiol. 80, 4511–4518. 10.1128/AEM.00687-14 24837387 PMC4148786

[B22] Ji X. Xu Y. Zhang C. Chen N. Lu X. (2012). A new locus affects cell motility, cellulose binding, and degradation by *Cytophaga hutchinsonii*. Appl. Microbiol. Biotechnol. 96, 161–170. 10.1007/s00253-012-4051-y 22543350

[B23] Jones D. T. (1999). Protein secondary structure prediction based on position-specific scoring matrices. J. Mol. Biol. 292, 195–202. 10.1006/jmbi.1999.3091 10493868

[B24] Jun H. S. Qi M. Gong J. Egbosimba E. E. Forsberg C. W. (2007). Outer membrane proteins of Fibrobacter succinogenes with potential roles in adhesion to cellulose and in cellulose digestion. J. Bacteriol. 189, 6806–6815. 10.1128/JB.00560-07 17644604 PMC2045214

[B25] Korotkov K. V. Hol W. G. (2008). Structure of the GspK-GspI-GspJ complex from the enterotoxigenic *Escherichia coli* type 2 secretion system. Nat. Struct. Mol. Biol. 15, 462–468. 10.1038/nsmb.1426 18438417

[B26] Korotkov K. V. Sandkvist M. Hol W. G. (2012). The type II secretion system: biogenesis, molecular architecture and mechanism. Nat. Rev. Microbiol. 10, 336–351. 10.1038/nrmicro2762 22466878 PMC3705712

[B27] Larkin J. (1989). Nonphotosynthetic, nonfruiting gliding bacteria, in Bergey's Manual of Systematic Bacteriology, eds Staley J. T. Bryant M. P. Pfennig N. Holt J. G. (Baltimore, MD: Williams and Wilkins), 2010–2138.

[B28] Larkin M. A. Blackshields G. Brown N. P. Chenna R. McGettigan P. A. McWilliam H.. (2007). Clustal, W., and Clustal X version 2.0. Bioinformatics 23, 2947–2948. 10.1093/bioinformatics/btm404 17846036

[B29] Le Blastier S. Hamels A. Cabeen M. Schille L. Tilquin F. Dieu M.. (2010). Phosphate starvation triggers production and secretion of an extracellular lipoprotein in Caulobacter crescentus. PLoS ONE 5:e14198. 10.1371/journal.pone.0014198 21152032 PMC2996285

[B30] Li L. Y. Shoemaker N. B. Salyers A. A. (1995). Location and characteristics of the transfer region of a *Bacteroides* conjugative transposon and regulation of transfer genes. J. Bacteriol. 177, 4992–4999. 10.1128/jb.177.17.4992-4999.1995 7665476 PMC177276

[B31] Lynd L. R. Weimer P. J. van Zyl W. H. Pretorius I. S. (2002). Microbial cellulose utilization: fundamentals and biotechnology. Microbiol. Mol. Biol. Rev. 66, 506–577. 10.1128/MMBR.66.3.506-577.2002 12209002 PMC120791

[B32] McBride M. J. Xie G. Martens E. C. Lapidus A. Henrissat B. Rhodes R. G.. (2009). Novel features of the polysaccharide-digesting gliding bacterium *Flavobacterium johnsoniae* as revealed by genome sequence analysis. Appl. Environ. Microbiol. 75, 6864–6875. 10.1128/AEM.01495-09 19717629 PMC2772454

[B33] McBride M. J. Zhu Y. (2013). Gliding motility and Por secretion system genes are widespread among members of the phylum bacteroidetes. J. Bacteriol. 195, 270–278. 10.1128/JB.01962-12 23123910 PMC3553832

[B34] Nakane D. Sato K. Wada H. Mcbride M. J. Nakayama K. (2013). Helical flow of surface protein required for bacterial gliding motility. Proc. Natl. Acad. Sci. U.S.A. 110, 11145–11150. 10.1073/pnas.1219753110 23781102 PMC3704026

[B35] Nivaskumar M. Francetic O. (2014). Type II secretion system: a magic beanstalk or a protein escalator. Biochim. Biophys. Acta 8:3. 10.1016/j.bbamcr.2013.12.020 24389250

[B36] Peabody C. R. Chung Y. J. Yen M. R. Vidal-Ingigliardi D. Pugsley A. P. Saier M. H. Jr. (2003). Type II protein secretion and its relationship to bacterial type IV pili and archaeal flagella. Microbiology 149(Pt 11), 3051–3072. 10.1099/mic.0.26364-0 14600218

[B37] Reichenbach H. (2006). The Order Cytophagales. New York, NY: Springer.

[B38] Rondelet A. Condemine G. (2013). Type II secretion: the substrates that won't go away. Res. Microbiol. 164, 556–561. 10.1016/j.resmic.2013.03.005 23538405

[B39] Rossier O. Starkenburg S. R. Cianciotto N. P. (2004). Legionella pneumophila type II protein secretion promotes virulence in the A/J mouse model of Legionnaires' disease pneumonia. Infect. Immun. 72, 310–321. 10.1128/IAI.72.1.310-321.2004 14688110 PMC344012

[B40] Sandkvist M. (2001). Type II secretion and pathogenesis. Infect. Immun. 69, 3523–3535. 10.1128/IAI.69.6.3523-3535.2001 11349009 PMC98326

[B41] Shrivastava A. Johnston J. J. van Baaren J. M. McBride M. J. (2013). *Flavobacterium johnsoniae* GldK, GldL, GldM, and SprA are required for secretion of the cell surface gliding motility adhesins SprB and RemA. J. Bacteriol. 195, 3201–3212. 10.1128/JB.00333-13 23667240 PMC3697645

[B42] Sikora A. E. Lybarger S. R. Sandkvist M. (2007). Compromised outer membrane integrity in Vibrio cholerae type II secretion mutants. J. Bacteriol. 189, 8484–8495. 10.1128/JB.00583-07 17890307 PMC2168955

[B43] Soding J. Biegert A. Lupas A. N. (2005). The HHpred interactive server for protein homology detection and structure prediction. Nucleic Acids Res. 33, W244–W248. 10.1093/nar/gki408 15980461 PMC1160169

[B44] Stanier R. Y. (1942). Agar-decomposing strains of the Actinomyces Coelicolor species-group. J. Bacteriol. 44, 555–570. 16560595 10.1128/jb.44.5.555-570.1942 PMC373707

[B45] Vignon G. Kohler R. Larquet E. Giroux S. Prevost M. C. Roux P.. (2003). Type IV-like pili formed by the type II secreton: specificity, composition, bundling, polar localization, and surface presentation of peptides. J. Bacteriol. 185, 3416–3428. 10.1128/JB.185.11.3416-3428.2003 12754241 PMC155369

[B46] Wang Y. Wang Z. Cao J. Guan Z. Lu X. (2014). FLP-FRT-based method to obtain unmarked deletions of CHU_3237 (porU) and large genomic fragments of *Cytophaga hutchinsonii*. Appl. Environ. Microbiol. 80, 6037–6045. 10.1128/AEM.01785-14 25063660 PMC4178665

[B47] Wilson D. B. (2008). Three microbial strategies for plant cell wall degradation. Ann. N. Y. Acad. Sci. 1125, 289–297. 10.1196/annals.1419.026 18378599

[B48] Xie G. Bruce D. C. Challacombe J. F. Chertkov O. Detter J. C. Gilna P.. (2007). Genome sequence of the cellulolytic gliding bacterium *Cytophaga hutchinsonii*. Appl. Environ. Microbiol. 73, 3536–3546. 10.1128/AEM.00225-07 17400776 PMC1932680

[B49] Xu Y. Ji X. Chen N. Li P. Liu W. Lu X. (2012). Development of replicative oriC plasmids and their versatile use in genetic manipulation of *Cytophaga hutchinsonii*. Appl. Microbiol. Biotechnol. 93, 697–705. 10.1007/s00253-011-3572-0 21935590

[B50] Zhang C. Wang Y. Li Z. Zhou X. Zhang W. Zhao Y.. (2014). Characterization of a multi-function processive endoglucanase CHU_2103 from *Cytophaga hutchinsonii*. Appl. Microbiol. Biotechnol. 98, 6679–6687. 10.1007/s00253-014-5640-8 24652064

[B51] Zhou H. Wang X. Yang T. Zhang W. Chen G. Liu W. (2015). Identification and characterization of a novel locus in *Cytophaga hutchinsonii* involved in colony spreading and cellulose digestion. Appl. Microbiol. Biotechnol. 99, 4321–4331. 10.1007/s00253-015-6412-9 25661809

[B52] Zhou H. Wang X. Yang T. Zhang W. Chen G. Liu W. (2016). An outer membrane protein involved in the uptake of glucose is essential for *Cytophaga hutchinsonii* cellulose utilization. Appl. Environ. Microbiol. 82, 1933–1944. 10.1128/AEM.03939-15 26773084 PMC4784033

[B53] Zhu Y. Han L. Hefferon K. L. Silvaggi N. R. Wilson D. B. McBride M. J. (2016). Periplasmic *Cytophaga hutchinsonii* endoglucanases are required for use of crystalline cellulose as the sole source of carbon and energy. Appl. Environ. Microbiol. 82, 4835–4845. 10.1128/AEM.01298-16 27260354 PMC4984284

[B54] Zhu Y. Li H. Zhou H. Chen G. Liu W. (2010). Cellulose and cellodextrin utilization by the cellulolytic bacterium *Cytophaga hutchisonii*. Bioresour. Technol. 101, 6432–6437. 10.1016/j.biortech.2010.03.041 20362433

[B55] Zhu Y. McBride M. J. (2014). Deletion of the *Cytophaga hutchinsonii* type IX secretion system gene sprP results in defects in gliding motility and cellulose utilization. Appl. Microbiol. Biotechnol. 98, 763–775. 10.1007/s00253-013-5355-2 24257839

[B56] Zhu Y. Zhou H. Bi Y. Zhang W. Chen G. Liu W. (2013). Characterization of a family 5 glycoside hydrolase isolated from the outer membrane of cellulolytic *Cytophaga hutchinsonii*. Appl. Microbiol. Biotechnol. 97, 3925–3937. 10.1007/s00253-012-4259-x 22790541

