# Corrigendum: A Putative Type II Secretion System Is Involved in Cellulose Utilization in *Cytophaga hutchisonii*

**DOI:** 10.3389/fmicb.2017.01758

**Published:** 2017-09-15

**Authors:** Xia Wang, Qingqing Han, Guanjun Chen, Weixin Zhang, Weifeng Liu

**Affiliations:** State Key Laboratory of Microbial Technology, School of Life Science, Shandong University Jinan, China

**Keywords:** *Cytophaga hutchisonii*, cellulose degradation, cellulose adhesion, protein secretion, T2SS

In the original article Zhu et al. (2017) was not cited in the article. The citation has now been inserted in Materials and Methods, Plasmids Constructions, Paragraph 1 and Table 1 and should read:

**Table 1:** Strains and plasmids used in this study.

**Strains and plasmids**	**Description**	**Reference or source**
**STRAINS**		
*C. hutchinsonii*	Wild type	ATCC
ATCC 33406		
Δ3195	Targeted insertion in *chu_3195*; *Em^*r*^*	This study
Δ3196	Targeted insertion in *chu_3196*; *Em^*r*^*	This study
Δ3198	Targeted deletion of *chu_3198*; *Em^*r*^*	This study
Δ3199	Targeted insertion in *chu_3199*; *Em^*r*^*	This study
Δ1253	Targeted insertion in *chu_1253*; *Em^*r*^*	This study
COM3195	Complemented strain with plasmid pCH3195; *Em^*r*^, Cm^*r*^*	This study
COM3199	Complemented strain with plasmid pCH3199; *Em^*r*^, Cm^*r*^*	This study
*E. coli* DH5α	F −φ80*lacZ*ΔM15 Δ*(lacZYA-argF)* U169 *recA1 endA1 hsdR17(rk^−^, mk^+^) phoA supE44 λ − thi-1 gyrA96 relA1*	Laboratory stock
**PLASMIDS^a^**		
pLYL03	ColE1; *Bacteroides*–*Flavobacterium* suicide vector; *Ap^*r*^* (*Em^*r*^*)	Li et al., 1995
pYT313	*sacB*-containing suicide vector; *Ap^*r*^ (Em^*r*^)*	Zhu et al., 2017
pLYIN3195	pLYL03 carrying an 1.0-kbp internal fragment of *chu_1719*; *Ap^*r*^* (*Em^*r*^*)	This study
pYT3198	PYT313 carrying two 2.0-kbp fragments upstream and downstream of *chu_3198*; *Ap^*r*^* (*Em^*r*^*)	This study
pLYIN3199	pLYL03 carrying an 748-bp internal fragment of *chu_3196*; *Ap^*r*^* (*Em^*r*^*)	This study
pLYIN1253	pLYL03 carrying an 825-bp internal fragment of *chu_1253*; *Ap^*r*^* (*Em^*r*^*)	This study
pCH03C	pLYL03oriC containing *cat* resistant gene; *Ap^*r*^* (*Em^*r*^, Cm^*r*^*)	Zhou et al., 2016
pCH3195	pCH03C containing an expression cassette of *chu_3195* under control of the *chu_1284* promoter; *Ap^*r*^* (*Em^*r*^, Cm^*r*^*)	This study
pCH3198	pCH03C containing an expression cassette of *chu_3198* under control of the *PompA* promoter; *Ap^*r*^* (*Em^*r*^, Cm^*r*^*)	This study
pCH3199	pCH03C containing an expression cassette of *chu_3199* under control of the *chu_1284* promoter; *Ap^*r*^* (*Em^*r*^, Cm^*r*^*)	This study

a *Antibiotic resistance phenotypes: ampicillin (Apr), chloramphenicol (Cmr), erythromycin (Emr), kanamycin (Kmr). Unless indicated otherwise, the antibiotic resistance phenotypes are those expressed in E. coli. The antibiotic resistance phenotypes in parentheses are expressed in C. hutchinsonii*.

To generate the pYT3198 plasmid, two DNA fragments corresponding to approximately 2 kb of chu_3198 up- and downstream regions were amplified from *C. hutchinsonii* chromosomal DNA with the primers 3198upF/3198upR, and 3198downF/3198downR, respectively, and ligated into the pYT313 plasmid (Zhu et al., 2017).

The authors apologize for this error and state that this does not change the scientific conclusions of the article in any way.

## Conflict of interest statement

The authors declare that the research was conducted in the absence of any commercial or financial relationships that could be construed as a potential conflict of interest.
