# Supplementary material for: A Putative Type II Secretion System Is Involved in Cellulose Utilization in Cytophaga hutchisonii
Source: Front Microbiol. 2017 Aug 9;8:1482. doi: 10.3389/fmicb.2017.01482 (PMC5553014; doi:10.3389/fmicb.2017.01482)
Supplement: Supplementary file 1 [file DataSheet1.docx]

**A putative type II secretion system is involved in cellulose utilization in *Cytophaga hutchisonii***

Xia Wang, Qingqing Han, Guanjun Chen, Weixin Zhang*, Weifeng Liu

No.27 Shanda South Road, State Key Laboratory of Microbial Technology, School of Life Science, Shandong University, Jinan 250100, Shandong, P. R. China

**Supplemental data**

*Correspondence should be addressed to Weixin Zhang. Tel.:+86 531 88364324; Fax: +86 531 88565610; e-mail: zhangwx@sdu.edu.cn

**Supplemental Materials and Methods**

**RT-PCR analyses**

Total RNA was extracted from *C. hutchinsonii* cells using the TRIzol reagent (Invitrogen, Grand Island, NY, USA), followed by genomic DNA removal with TURBO DNA-free kit (Ambion, Austin, TX, USA). The purity of the isolated RNA was determined by the ratio of optical density OD_260_/_280_, which was in range of 1.8~2.1 for all samples and the integrity was evaluated via electrophoretic analysis of the 23S and 16S rRNA subunits. Reverse transcription was carried out using the PrimeScript RT reagent Kit (Takara, Japan) exactly following the manufacture’s instructions except prolonging of the incubation time for up to 1 h to synthesize cDNA fragments in longer length. PCR amplification was performed in 20 µl of reaction mixture with gradient annealing temperatures for optimal amplification using approximately 25 ng of cDNA as template.

**Table S1** Primers used in this study.

| **Primers** | **Sequences (5’ to 3’)** | **Description** |
| --- | --- | --- |
| 3195inF | CGGGATCCGGTTTTTATTGCTTCCCCTTTTG | Forward primer to amplify a 1.5-kbp internal fragment of *chu_3195* |
| 3195inR | GCTCTAGACTCTCAAGCCTTCAAGCTTAC | Reverse primer to amplify a 1.5-kbp internal fragment of *chu_3195* |
| 3199inF | CGGGATCCGCTGCGCTGCAGGGAGAAACAG | Forward primer to amplify a 1.0-kbp internal fragment of *chu_3199* |
| 3199inR | GCTCTAGACGCGTACCATACTTACTTTTGATG | Reverse primer to amplify a 1.0-kbp internal fragment of *chu_3199* |
| 1253inF | CGGGATCCCTATTGACATCTGCATCC | Forward primer to amplify a 0.82-kbp internal fragment of *chu_1253* |
| 1253inR | GCTCTAGACCTGAGTAATAATAATTTTTAT | Reverse primer to amplify a 0.82-kbp internal fragment of *chu_1253* |
| P3195F | ATGGTTAAAAAAATTTCCTG | Forward primer used in anchored PCR for verification of *chu_3195* inactivation |
| P3195R | TTAATAAATTATTATAGGTTTG | Reverse primer used in anchored PCR for verification of *chu_3195* inactivation |
| P3198F | ATGAAAAATATTTTATCCGCG | Forward primer used in anchored PCR for verification of *chu_3198* deletion and to amplify the *chu_3198* gene |
| P3198R | TTAATCTTTTACTGTTTCCTG | Reverse primer used in anchored PCR for verification of *chu_3198* deletion and to amplify the *chu_3198* gene |
| P3199F | GCAATAGAAATAAATAAAATTG | Forward primer used in anchored PCR for verification of *chu_3199* inactivation |
| P3199R | TTAAAATGAATTGCCTAGCTGG | Reverse primer used in anchored PCR for verification of *chu_3199* inactivation |
| P1253F | CGGGATCCATGATTAGTACATGTAAGTTTTTATC | Forward primer used in anchored PCR for verification of *chu_1253* inactivation |
| P1253R | GCTCTAGATTAGAAAATAAAGAATTGCAATTG | Reverse primer used in anchored PCR for verification of *chu_1253* inactivation |
| anchorF | CGAAAAAATCGCTATAATGAC | Forward primer within pLYL03 for anchored PCR |
| anchorR | GTGGAATTGTGAGCGGATAAC | Reverse primer within pLYL03 for anchored PCR |
| ermFF | ATGACAAAAAAGAAATTGCCCGTTCG | Forward primer to amplify the *ermF* gene |
| ermFR | AGGGACAACTTCCAGCATTTCC | Reverse primer to amplify the *ermF* gene |
| oriCF | AACTGCAGTATATATCATTTCTTCTATTATAATT | Forward primer to amplify the *oriC* sequence |
| oriCR | ACGCGTCGACATATTTTAAACAGCATTTATTAAC | Reverse primer to amplify the *oriC* sequence |
| P1284F | CGGGATCCTGTTTGATGTAGTTAGATATTA | Forward primer to amplify the *chu_1284* promoter sequence |
| P1284-3195R | GAAATAAGAAAAGTTTTATTTATGGTTAAAAAAATTTCCTG | Reverse primer to amplify the *chu_1284* promoter fused to *chu_3195* gene |
| P1284-3195F | CAGGAAATTTTTTTAACCATAAATAAAACTTTTCTTATTTC | Forward primer to amplify the *chu_3195* gene for fusion PCR |
| 3195R | GCTCTAGATTAATAAATTATTATAGGTTTG | Reverse primer to amplify the *chu_3195* gene |
| P1284-3199F | GAAATAAGAAAAGTTTTATTTATGGCAATAGAAATAAATAAAATTG | Forward primer to amplify the *chu_3199* gene for fusion PCR |
| P1284-3199R | CAATTTTATTTATTTCTATTGCCATAAATAAAACTTTTCTTATTTC | Reverse primer to amplify the *chu_1284* promoter fused to *chu_3199* gene |
| 3199R | GCTCTAGATTAAAATGAATTGCCTAGCTGG | Reverse primer to amplify the *chu_3199* gene |
| PompA-3198F | CGGGATCCTTGCCACATTTGGTGTTTTTTTGTAG | Forward primer to amplify the *chu_3198* gene for fusion PCR |
| PompA-3198R | CTCGCGGATAAAATATTTTTCATACTTAATTTTTTTAATTACAATTTAG | Reverse primer to amplify the *chu_1284* promoter fused to *chu_3199* gene |
| 3198R | GCTCTAGATTAATCTTTTACTGTTTCCTG | Reverse primer to amplify the *chu_3198* gene |
| 3198F-anchor | GTAAATAAAGTAGATCGTTTTTTAG | Forward primer for anchored PCR in *chu_3198* |
| 3198R-anchor | CATAGAGCAAAACAATCAGCAG | Reverse primer for diagnostic anchored PCR in *chu_3198* |
| 3198upF | GCAGCGGAAAAATTCGGGGGATCCGATTGCGATGCTGATCATCTGTG | Forward primer to amplify a 2.0-kbp fragment upstream of *chu_3198* |
| 3198upR | CAGCAGTACATGAATTGTCATGTAAATTAATAAACTATAATGTTATAAAATTAC | Reverse primer to amplify a 2.0-kbp fragment upstream of *chu_3198* |
| 3198downF | TACATGACAATTCATGTACTGCTG | Forward primer to amplify a 2.0-kbp fragment downstream of *chu_3198* |
| 3198downR | CAAGCTTGCATGCCTGCAGGTCGACCATCTAACGATGTGTTGCCTTC | Reverse primer to amplify a 2.0-kbp fragment downstream of *chu_3198* |

Note, Restriction enzyme sites in primers are underlined.

**Table S2** Mass spectroscopic identification of missing or weakened OMPs in *t2s* mutant strains shown in Figure 5.

|  | **Matched peptides (in bold)** | **Targeted protein** | **Score^a^** |
| --- | --- | --- | --- |
| 1 | MVSNAQVVYW NGLGRALVTG SYLNGNILKP YSDSTDQANI VTQYKDSTSA RKSTDGYTIF DLGVNAQPNE ALRASATLRL SNSFGGFYGD GSQFIFRQLR LDGIIGKKVK YEIGDIDLEL SKYTIFNSNE IYNDYESDII AQRRSVVEYE NFNFGNKWR**L QGAHIETGLR** FEQGIEKLGL RAFATRNRRY TPSITPDRYM MGGRVELVQS RMLQVGGNYV YIFDAAGTVS SPGLTNQNQV LTGDWKVTHY MDKIDLSFYG EAGKSNNKYA VIEKDSVKTD DYFYDLGLSA KYKPWLLKLF VNYR**NVGADF FSSGAQTR**RV NDYGYNGPHG NNTLGMFDQV QNNAVQR**ATI GGATLLDYVS DQNLR**NLNLK NTLMAFNPAY NNITPYGQAT ANRKGLTIGA SLGNAEKVVK ADFVADLLSE VASQGDTVDN ALRKFTGLKG GAMVNIHKLL RFEK**NIIFTA GAR**YEKTTRG GVSPVDLQST LIDLGLTVEV VKNLDLIGGI KTITAKGNEY IYVR**NDFNQI R**TDNPFSNYK **LDQSQFLSTF GAR**YR**FSKNT YFTVNGVTQS VK**FDDASYNN YKINQIFFNY TMIF | hypothetical protein CHU_1277 | 56 |
| 2 | MLLTSASFAN GPLTTNEEDP IKKNAKSKQA KNGSEVFKTM QKTPKNKYIL REVPGYFFET GTEATAQAAA DTTPFKPSIS AGAIVHMMGT YSQAGFGPGT NSSDQWNK**GF MLYR**ARILLG GQLSK**KGSFF METEVPSAIG TR**IDSTTKNV KVSPIILDCQ YEHK**FSTALT LIGGMQLVSH NR**NGLQGAAS LLANDFTFFQ YPYNLFENQP LQGNFGRDLG VNAR**GYFLNE K**LEYRIGVFT GRRATDNSPL RTVGR**VVYNF LDADKNYYYS GTNLGNGK**TI SLGAGFDAQG SYSNIGADLF VDMPAGNPGS VTLSAAFSYM TGGTSLTATN SFARLIPK**QT TQLLELGYYF K**DIKLQPYIR **YENQSINAED NQVFGTTDK**S TFNKLNSSSV FGGGLNYFFN GYGTNLRLSY TSFTKGVLNT SGEVDKKSFG QIWLQLQFFI F | hypothetical protein CHU_1253 | 89 |
| 3 | MRHGKK**DNHL GRTVTHR**RAL LSNMASSLIL HKRISTTVPK AKELRKYVEP LITKAKADST HSRR**TVFSYL QNKDSVK**ELF DNVVEKIAAR PGGYTRILKT GARIGDNADM CIIELVDYNE TYVKDSEVKV AAKRTRRSTA AKKDDSSEST GAEPQA | 50S ribosomal protein L17（CHU_3135） | 34 |
| 4 | MR**LALDAMGG DFAPK**AIIEG AILASDSLPA DAEIILIGKE QLIR**EHLSTL EGNK**SRIQIV HADEVIGMAE HPTKAFSQKP NSSIAVGFHL LKQKEVDAFC SAGNTGAMLV GSMFSIKPIE GILRPGIAGF MPQESGRFAV VIDVGANADC KPEMLAQFAV IGSLYTKYVF GIENPKVGLM NLGEEEEKGT ILTLAAHQLI KELDSINFIG NIEGRDLFNN KADVIVCDGY TGNIILK**MGE TFYDLVSK**RG YDDTFFNMFN YEEVGGSPIL GVNGNVIIGH GISSPKAIKN MMHQAYQLTQ AKISEKIKNA YS | phosphate acyltransferase (CHU_1625) | 24 |
| 5 | MQIIEKYFKH LSPEQVKQFG ALHSLYTEWN EKINVVSR**KD IDQLYER**HVL HSLGIAKVMA FKPGTR**ILDV GTGGGFPGIP LSILFPESDF HLIDSIGK**KI KVVEEVSAGA GIKNIRTTHG RAEDVTDRYH FVVSRAVTRF KPFWGWVAKK FSDEQFNDLN NGILYLKGGD LDEEIQELNR PAYEYDLNMF FEEEFFDTKK VVHVPAF | ribosomal RNA small subunit methyltransferase G (CHU_1194) | 21 |
| 6 | MNK**LLMLALA AATIVISYAQ NAPGTAQKGA VTVNPK**DVEA GKALVAQSDC KTCHVSDYTL IGPGYKQIAQ RYPLTETNIQ YLSKKIISGG GGVWGENEMA AHTTLTPEQT RKIALYILSL K | cytochrome c551/c552 (CHU_0559) | 21 |
| 7 | M**NKEIILHIK** NMVCPRCIKV VTDELTELGI NTINVSLGKV QLTAPVSNES LFEIKKALEK NGFELLDDKK SKVIEQIKIL IIEGIRDGKF SEMHINLSQY INDQVQMEYS YLSNLFSSVE GKTIER**FVIL QKIER**IKELI SYDELSIKEI AGLLGYSSLQ ALSNQFKKET GMNPRDFKKI SADVTRNPIN DI | transcriptional regulator, AraC family ( CHU_1500) | 15 |
| 8 | MSTGIQYIII GAIVGWAGVY LFRKIYGLFN VPK**DGCGGGC GCASAELKK**EKK | hypothetical protein CHU_3260 | 21 |

Note, *^a^*Score is -10*Log(P), where P is the probability that the observed match is a random event. Scores greater than 52 are significant (*p*<0.05).


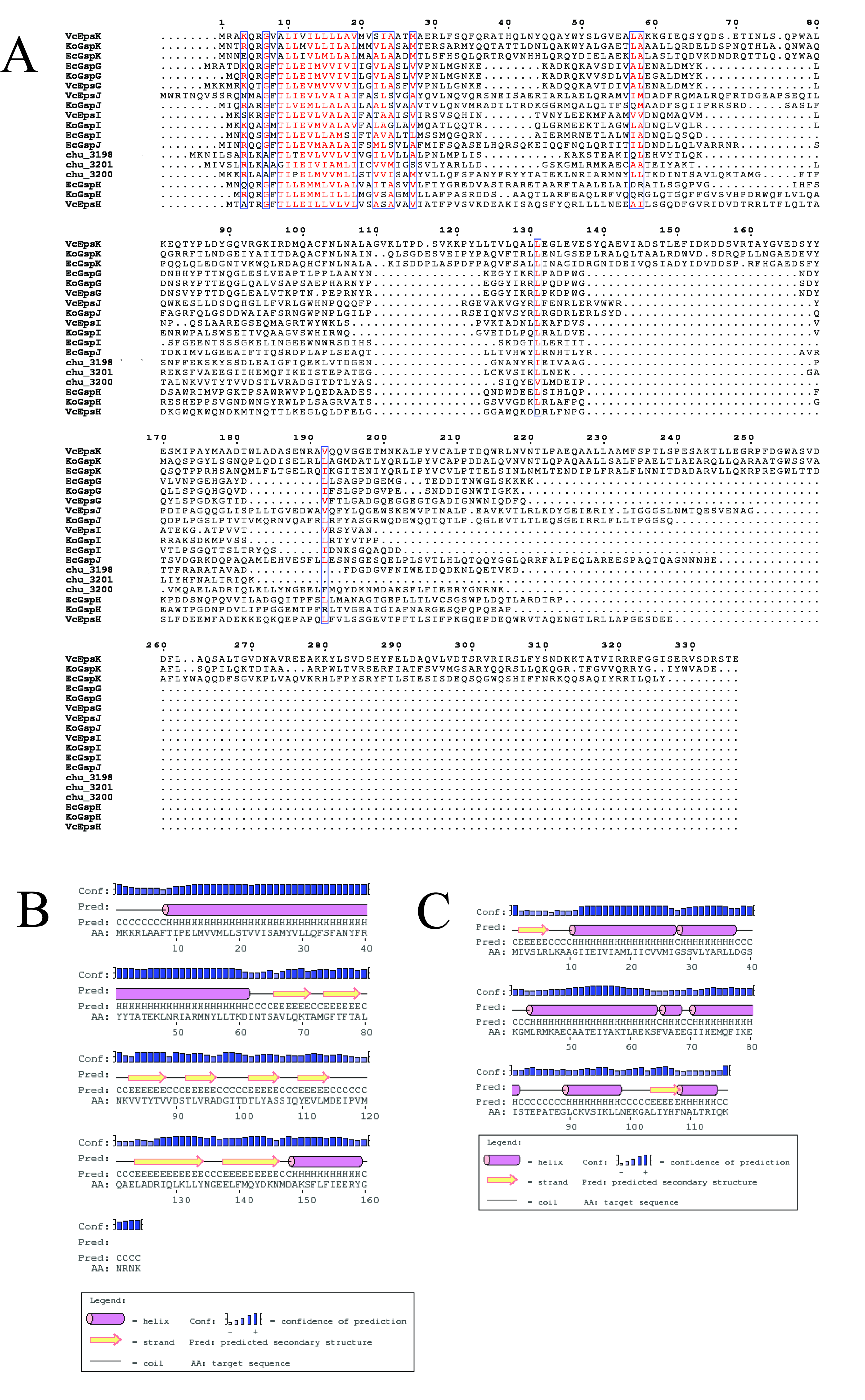


Figure S1 Multiple sequence alignment of CHU_3200, CHU_3201, and CHU_3198 (T2S-G) with well-characterized pseudopilins from *Escherichia coli* (*Ec*), *Klebsiella oxytoca* (*Ko*), and *Vibrio cholera* (*Vc*) (A) and the secondary structural analyses of CHU_3200 (B) and CHU_3201 (C). The sequences were retrieved from NCBI or KEGG database and sequence alignments were performed with Clustal W (Larkin et al., 2007). Secondary structural analyses were carried out using PSIPRED Server online (<http://bioinf.cs.ucl.ac.uk/psipred/>) (Jones, 1999).


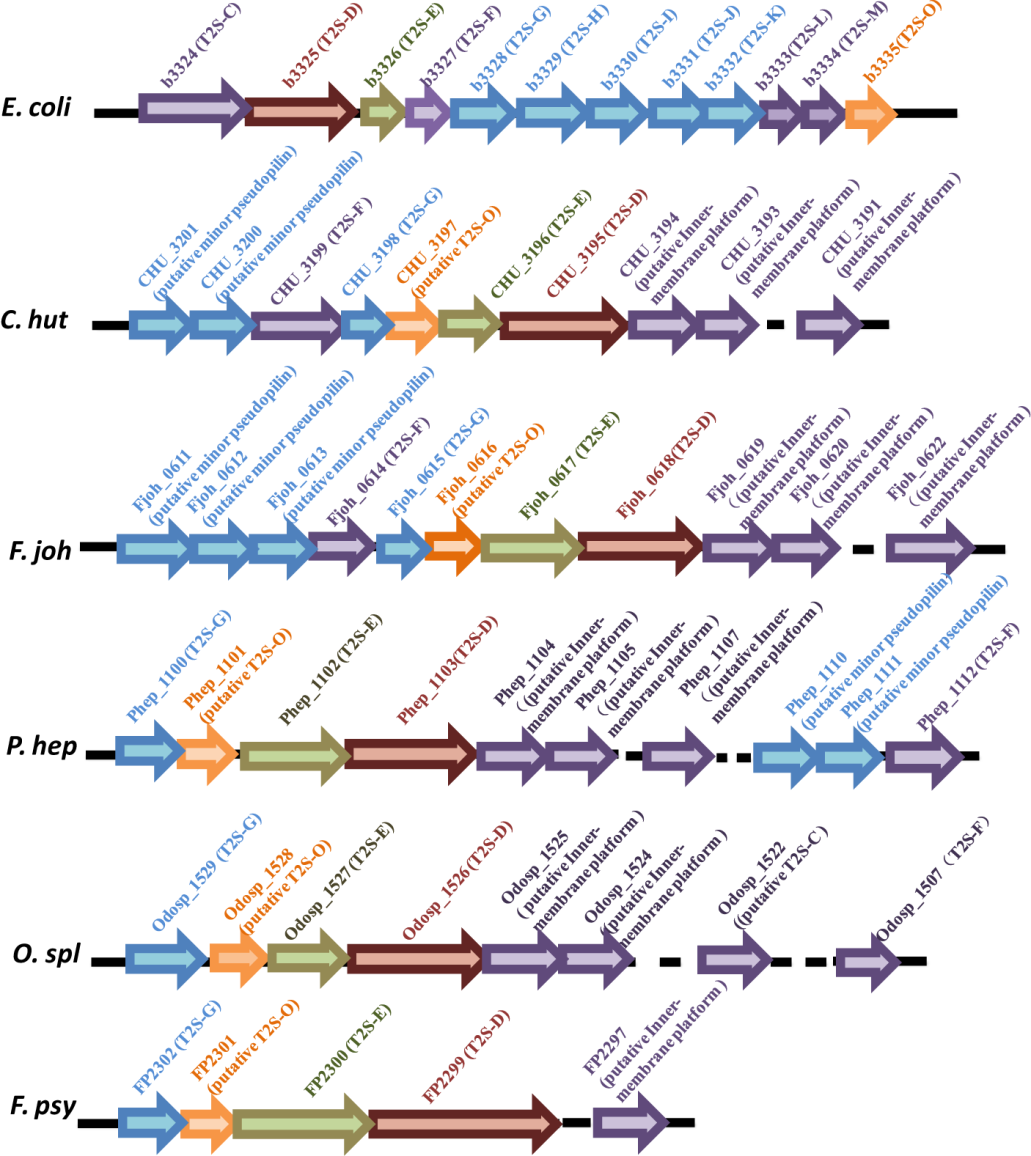


Figure S2 Schematic illustration of the organization of the putative *t2s* gene loci in genomes of the indicated bacteria. *E. coli*, *Escherichia coli* K12 (Hayashi et al., 2006); *C. hut*, *Cytophaga hutchinsonii* ATCC 33406 (Xie et al., 2007); *F. joh*, *Flavobacterium johnsoniae* (McBride et al., 2009); *F. psy*, *Flavobacterium psychrophilum* (Duchaud et al., 2007); *P. hep*, *Pedobacter heparinus* (Han et al., 2009); *O. spl*, *Odoribacter splanchnicus* (Goker et al., 2011).


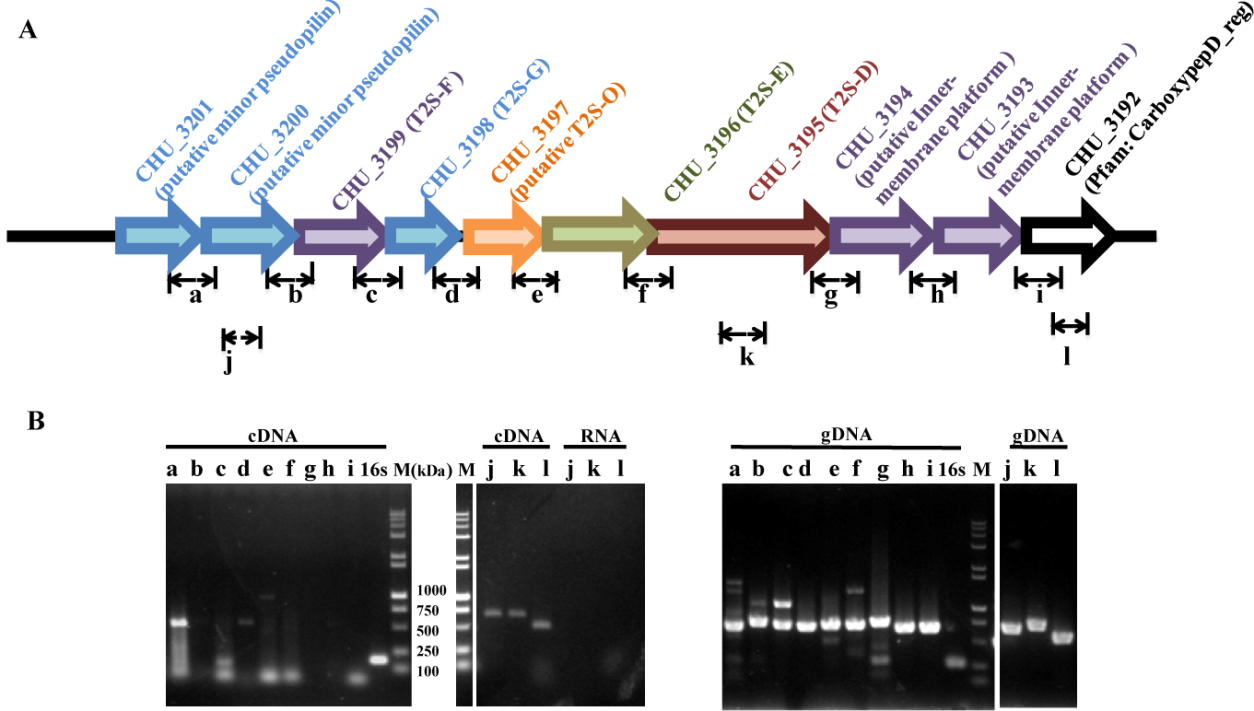


Figure S3 PCR amplification of the regions spanning two adjacent genes in the putative *t2s* gene cluster with cDNA from WT cells as template. (A) Schematic illustration of the loci and organization of *t2s* genes. Fragments denoted with a, b, c, d, e, f, g, h, and i represented those spanning the ends of two adjacent genes, respectively, whereas fragments denoted with j, k, and l indicated the internal regions of the indicated genes to be amplified, respectively. (B) PCR amplification of the regions as indicated in (A) with cDNA, gDNA, or total RNA as template, respectively. Only two fragments with expected size and correct sequence in lanes a and d (denoted with arrows) were amplified with cDNA as template, indicating that the majority of the identified putative *t2s* genes are most probably transcribed independently and thus do not fell into an operon. The successful amplification of all the spanning fragments with gDNA and of the three internal regions with cDNA as template (denoted with red square) verified the validity of primers and cDNA used in this assay. No amplified products were observed with extracted RNA as template, demonstrating that cDNA templates were free from gDNA contamination.


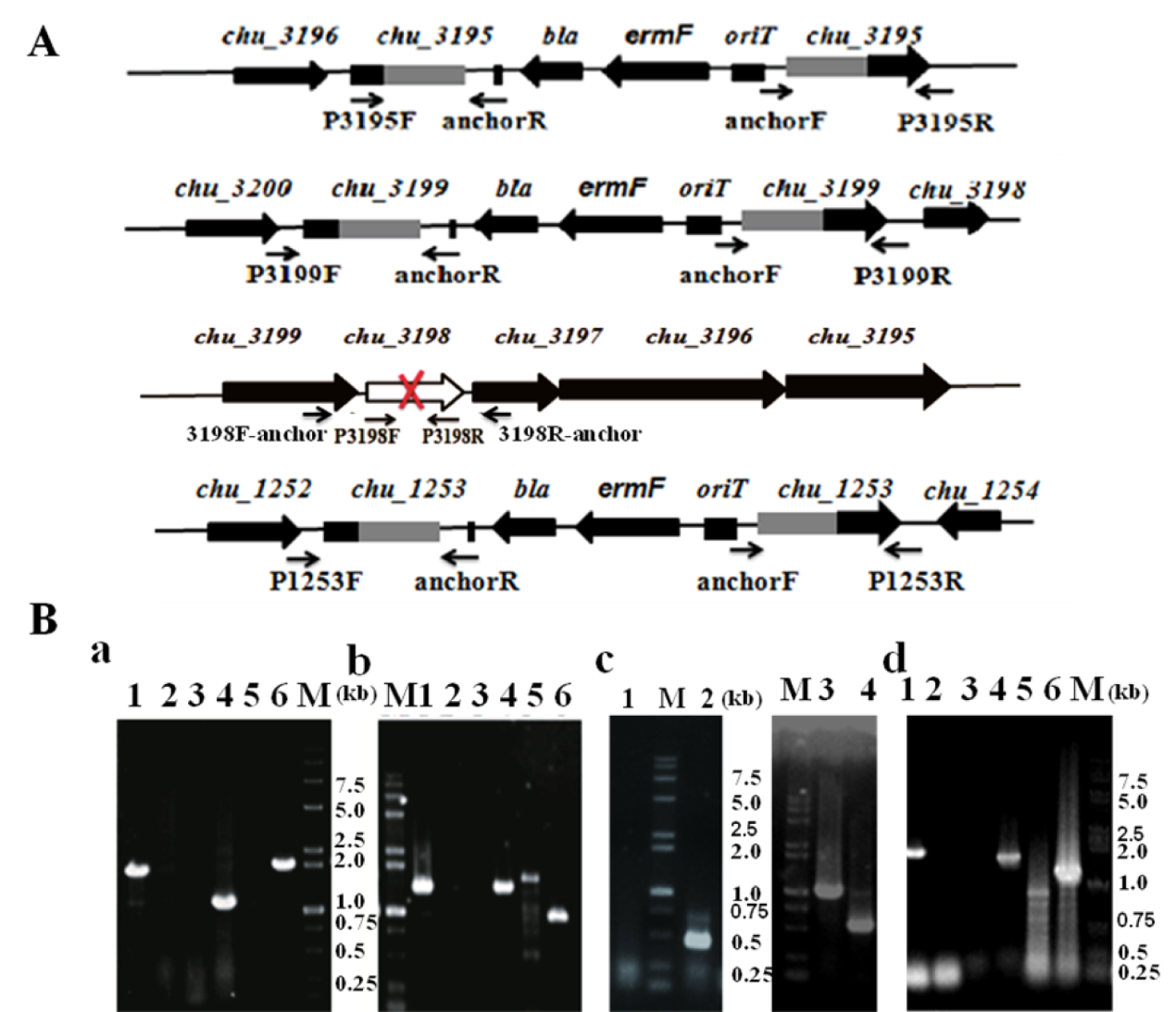


Figure S4 Verification of the targeted insertional disruptions of *chu_3195*, *chu_3199* and *chu_1253* and deletion of *chu_3198*. (A) Schematic illustration of the *chu_3195*, *chu_3199*, *chu_3198*, and *chu_1253* locus interrupted by targeted insertion of pLYIN3195, PYT3198, pLYIN3199, and PLYIN1253, respectively. (B) Anchored PCR analyses to verify the correct insertional inactivation of *chu_3195* (a), *chu_3199* (b), and *chu_1253* (d), and the deletion of *chu_3198* (c). (a, b, and d) Lane 1: PCR amplification of each gene using primers P3195F/P3195R, P3199F/P3199R, and P1253F/P1253R, respectively, with genomic DNA isolated from the individual mutant as template; Lane 2: PCR amplification of each gene using primers P3195F/P3195R, P3199F/P3199R, and P1253F/P1253R, respectively, with genomic DNA isolated from WT as template; Lane 3: PCR amplification using primers P3195F/anchorR, P3199F/anchorR, and P1253F/anchorR, respectively, with genomic DNA isolated from WT as template; Lane 4: PCR amplification using primers P3195F/anchorR, P3199F/anchorR, and P1253F/anchor, respectively, with genomic DNA isolated from the individual mutant as template; Lane 5: PCR amplification using primers anchorF/P3195R, anchorF/P3199R, and anchor/P1253R, respectively, with genomic DNA isolated from WT as template; Lane 6: PCR amplification using primers anchorF/P3195R, anchorF/P3199R, and anchor/P1253R, respectively, with genomic DNA isolated from the individual mutant as template. (c) Lanes 1 and 2: PCR amplification using primers P3198F and P3198R with genomic DNA isolated from WT and the Δ3198 transformant as template, respectively. Lanes 3 and 4: PCR amplification using the primers 3198F-anchor and 3198R-anchor with genomic DNA isolated from WT and Δ3198 transformant as template, respectively. All DNA products were amplified with the expected size.


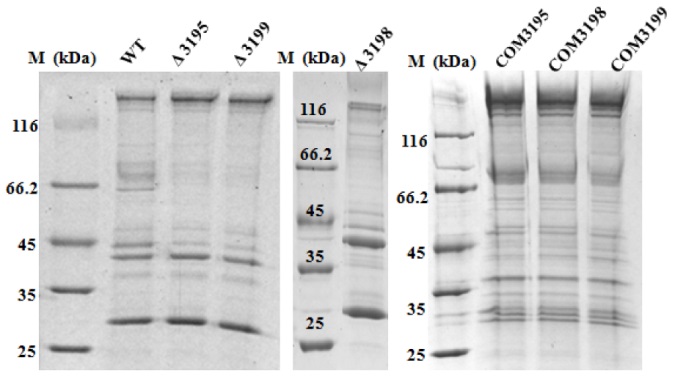


Figure S5 SDS-PAGE analysis of the cellulose-bound outer membrane proteins (OMPs) isolated from WT strain, the *t2s* mutant strain, and their individually complemented strains. Cells were cultured in PY10 medium supplemented with 0.4% glucose. Proteins were stained with Coomassie brilliant blue. Complementation of the mutant strains restored their compromised cellulose-bound OMP profile.


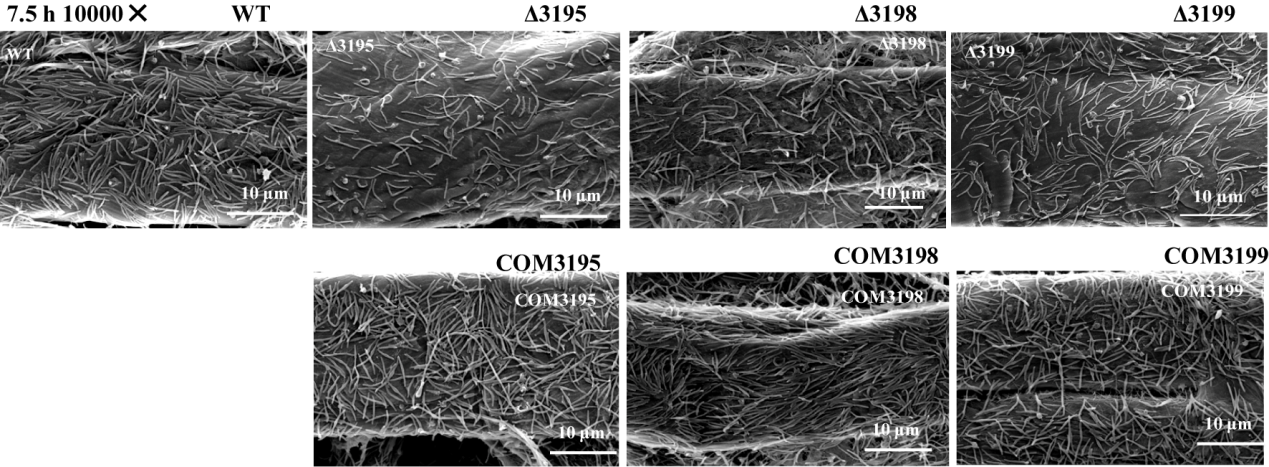


Figure S6 Scanning electron microscopic analyses of WT and mutant cells, as well as those complemented with the individual T2S component adhered to filter paper after incubation for 7.5 h. The respective complementation of the *t2s* mutants restored the bacterial-adhering capabilities of the mutant strains.


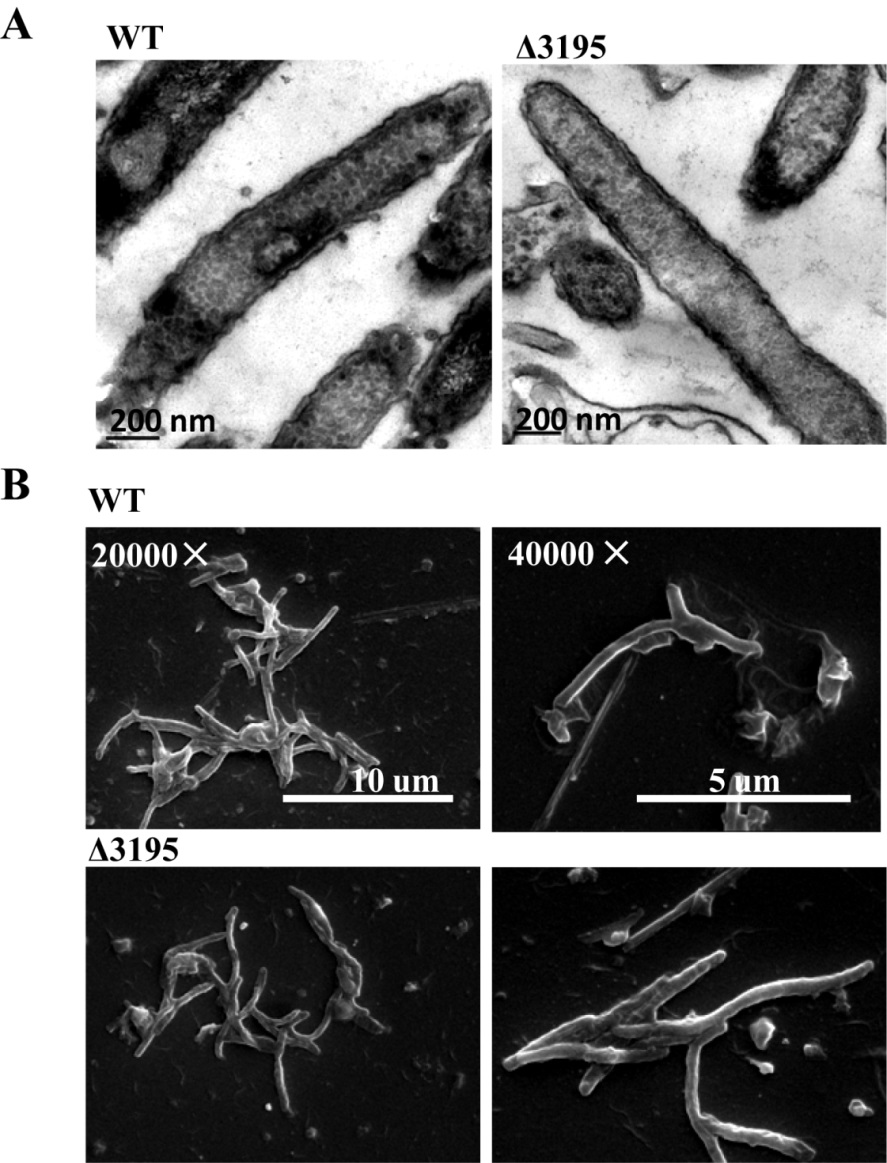


Figure S7 Morphological analyses of WT and the *chu_3195*-inactivated mutant cells using transmission (A) and scanning (B) electron microscopic analyses, respectively. Cells used in this assay were grown to mid-exponential phase in PY10 medium supplemented with 0.4% glucose. Hardly any difference in cell morphology was observed between WT and the *chu_3195*-inactivated strains.

**References**

Duchaud, E., Boussaha, M., Loux, V., Bernardet, J.F., Michel, C., Kerouault, B., et al. (2007). Complete genome sequence of the fish pathogen Flavobacterium psychrophilum. Nat Biotechnol 25(7), 763-769. doi: 10.1038/nbt1313.

Goker, M., Gronow, S., Zeytun, A., Nolan, M., Lucas, S., Lapidus, A., et al. (2011). Complete genome sequence of Odoribacter splanchnicus type strain (1651/6). Stand Genomic Sci 4(2), 200-209. doi: 10.4056/sigs.1714269.

Han, C., Spring, S., Lapidus, A., Del Rio, T.G., Tice, H., Copeland, A., et al. (2009). Complete genome sequence of Pedobacter heparinus type strain (HIM 762-3). Stand Genomic Sci 1(1), 54-62. doi: 10.4056/sigs.22138.

Hayashi, K., Morooka, N., Yamamoto, Y., Fujita, K., Isono, K., Choi, S., et al. (2006). Highly accurate genome sequences of Escherichia coli K-12 strains MG1655 and W3110. Mol Syst Biol 2, 2006 0007. doi: 10.1038/msb4100049.

Jones, D.T. (1999). Protein secondary structure prediction based on position-specific scoring matrices. J Mol Biol 292(2), 195-202. doi: 10.1006/jmbi.1999.3091.

Larkin, M.A., Blackshields, G., Brown, N.P., Chenna, R., McGettigan, P.A., McWilliam, H., et al. (2007). Clustal W and Clustal X version 2.0. Bioinformatics 23(21), 2947-2948. doi: 10.1093/bioinformatics/btm404.

Mcbride, M.J., Xie, G., Martens, E.C., Lapidus, A., Henrissat, B., Rhodes, R.G., et al. (2009). Novel Features of the Polysaccharide-Digesting Gliding Bacterium Flavobacterium johnsoniae as Revealed by Genome Sequence Analysis. *Applied & Environmental Microbiology* 75(21)**,** 6864-6875.

Xie, G., Bruce, D.C., Challacombe, J.F., Chertkov, O., Detter, J.C., Gilna, P., et al. (2007). Genome sequence of the cellulolytic gliding bacterium Cytophaga hutchinsonii. *Appl Environ Microbiol* 73(11)**,** 3536-3546.

**Supplemental movie S1 Cell motility of *C****. h****utchinsonii* cells.** Cells harvested from glucose culture were diluted in MMC buffer (10 mM MOPS, 4 mM MgSO4, 2 mM CaCl2, pH 7.6) to ~106 cell/ml. Five microliter of the suspension was transferred onto glass slides, and then were overlaid with about 200 μl of 1% methylcellulose in MMC buffer and placed at room temperature for 1 h. The motility of *C. hutchinsonii* cells was monitored with inverted phase contrast microscope (Nikon Eclipse TE2000-S), and continuous images were recorded at 1 s intervals. The images were analyzed using the ImageJ software, and Microsoft videos (7 frames/s, wmv file) were exported resulting in a 7×faster speed than real-time replay.

**Supplemental movie S2 Cell motility of the *chu_3195*-disrupted mutant cells.** Cells harvested from glucose culture were diluted in MMC buffer (10 mM MOPS, 4 mM MgSO4, 2 mM CaCl2, pH 7.6) to ~106 cell/ml. Five microliter of the suspension was transferred onto glass slides, and then were overlaid with about 200 μl of 1% methylcellulose in MMC buffer and placed at room temperature for 1 h. The motility of cells was monitored with inverted phase contrast microscope (Nikon Eclipse TE2000-S), and continuous images were recorded at 1 s intervals. The images were analyzed using the ImageJ software, and Microsoft videos (7 frames/s, wmv file) were exported resulting in a 7×faster speed than real-time replay.

**Supplemental movie S3 Cell motility of the *chu_3198*-disrupted mutant cells.** Cells harvested from glucose culture were diluted in MMC buffer (10 mM MOPS, 4 mM MgSO4, 2 mM CaCl2, pH 7.6) to ~106 cell/ml. Five microliter of the suspension was transferred onto glass slides, and then were overlaid with about 200 μl of 1% methylcellulose in MMC buffer and placed at room temperature for 1 h. The motility of cells was monitored with inverted phase contrast microscope (Nikon Eclipse TE2000-S), and continuous images were recorded at 1 s intervals. The images were analyzed using the ImageJ software, and Microsoft videos (7 frames/s, wmv file) were exported resulting in a 7×faster speed than real-time replay.

**Supplemental movie S4 Cell motility of the *chu_3199*-disrupted mutant cells.** Cells harvested from glucose culture were diluted in MMC buffer (10 mM MOPS, 4 mM MgSO4, 2 mM CaCl2, pH 7.6) to ~106 cell/ml. Five microliter of the suspension was transferred onto glass slides, and then were overlaid with about 200 μl of 1% methylcellulose in MMC buffer and placed at room temperature for 1 h. The motility of cells was monitored with inverted phase contrast microscope (Nikon Eclipse TE2000-S), and continuous images were recorded at 1 s intervals. The images were analyzed using the ImageJ software, and Microsoft videos (7 frames/s, wmv file) were exported resulting in a 7×faster speed than real-time replay.
